# Type II Secretion Substrates of *Legionella pneumophila* Translocate Out of the Pathogen-Occupied Vacuole via a Semipermeable Membrane

**DOI:** 10.1128/mBio.00870-17

**Published:** 2017-06-20

**Authors:** Hilary K. Truchan, Harry D. Christman, Richard C. White, Nakisha S. Rutledge, Nicholas P. Cianciotto

**Affiliations:** a Department of Microbiology and Immunology, Northwestern University Feinberg School of Medicine, Chicago, Illinois, USA; b Department of Pathology, Northwestern University Feinberg School of Medicine, Chicago, Illinois, USA; University of Michigan—Ann Arbor

**Keywords:** *Acanthamoeba*, chitinase, galectin, *Legionella pneumophila*, *Legionella*-containing vacuole, macrophage, protease, type II secretion

## Abstract

*Legionella pneumophila* replicates in macrophages in a host-derived phagosome, termed the *Legionella-*containing vacuole (LCV). While the translocation of type IV secretion (T4S) effectors into the macrophage cytosol is well established, the location of type II secretion (T2S) substrates in the infected host cell is unknown. Here, we show that the T2S substrate ProA, a metalloprotease, translocates into the cytosol of human macrophages, where it associates with the LCV membrane (LCVM). Translocation is detected as early as 10 h postinoculation (p.i.), which is approximately the midpoint of the intracellular life cycle. However, it is detected as early as 6 h p.i. if ProA is hyperexpressed, indicating that translocation depends on the timing of ProA expression and that any other factors necessary for translocation are in place by that time point. Translocation occurs with all *L. pneumophila* strains tested and in amoebae, natural hosts for *L. pneumophila*. It was absent in murine bone marrow-derived macrophages and murine macrophage cell lines. The ChiA chitinase also associated with the cytoplasmic face of the LCVM at 6 h p.i. and in a T2S-dependent manner. Galectin-3 and galectin-8, eukaryotic proteins whose localization is influenced by damage to host membranes, appeared within the LCV of infected human but not murine macrophages beginning at 6 h p.i. Thus, we hypothesize that ProA and ChiA are first secreted into the vacuolar lumen by the activity of the T2S and subsequently traffic into the macrophage cytosol via a novel mechanism that involves a semipermeable LCVM.

## IMPORTANCE

Infection of macrophages and amoebae plays a central role in the pathogenesis of *L. pneumophila*, the agent of Legionnaires’ disease. We have previously demonstrated that the T2S system of *L. pneumophila* greatly contributes to intracellular infection. However, the location of T2S substrates within the infected host cell is unknown. This report presents the first evidence of a *L. pneumophila* T2S substrate in the host cell cytosol and, therefore, the first evidence of a non-T4S effector trafficking out of the LCV. We also provide the first indication that the LCV is not completely intact but is instead semipermeable and that this occurs in human but not murine macrophages. Given this permeability, we hypothesize that other T2S substrates and LCV lumenal contents can escape into the host cell cytosol. Thus, these substrates may represent a battery of previously unidentified effectors that can interact with host factors and contribute to intracellular infection by *L. pneumophila*.

## INTRODUCTION

*Legionella pneumophila* is a Gram-negative, facultative intracellular bacterium that thrives in fresh water environments where it survives free, as part of multispecies biofilms, and within amoebae, which are the main environmental replicative niche (1, 2). Humans become infected with *L. pneumophila* after inhalation of contaminated aerosols produced in man-made water systems. Within the infected lung, the bacteria replicate in alveolar macrophages in a process similar to that which occurs within amoebae to cause a life-threatening pneumonia known as Legionnaires’ disease (3). In the United States, there are >18,000 cases of the disease per year (4), and recent reports signal an increasing incidence of the disease, especially among the immunocompromised (5, 6). After entry into host cells, *L. pneumophila* avoids fusion with degradative lysosomes and remodels its phagosome into a replicative niche known as the *Legionella-*containing vacuole (LCV). Smooth vesicles, rough endoplasmic reticulum (ER), and mitochondria are recruited to the LCV beginning at 2 h postinfection (p.i.) (7, 8). The host GTPase Rab1 is well known for being recruited to the cytoplasmic face of the LCV during smooth vesicle recruitment (9, 10). Soon after, the bacteria begin to replicate within the LCV and eventually increase their numbers by 50-fold to 100-fold. Macrophage lysis is evident after 24 h, at which point the legionellae escape and initiate new rounds of infection (11).

Secreted bacterial proteins orchestrate many aspects of *L. pneumophila* pathogenesis (12). The Dot/Icm type IV secretion (T4S) system delivers >320 effectors from the bacterial cytosol directly into the host cytosol via an apparatus that extends across the bacterial cell wall and LCV membrane (LCVM) (2, 13–15). These effectors interact with a variety of host factors and contribute to the formation of the highly specialized LCVs. Thus, T4S mutants that cannot deliver effectors do not grow intracellularly (13). It has been shown that type II secretion (T2S) also has a major role in *L. pneumophila* pathogenesis (16, 17). T2S is a two-step process wherein proteins destined for secretion are first trafficked across the bacterial inner membrane and into the periplasm via the Sec pathway or the Tat pathway (18). In a second step, the proteins are recognized by the T2S apparatus and exit the cell through a dedicated outer membrane pore. In *L. pneumophila* clinical isolate 130b, T2S substrates number more than 25 and include degradative enzymes such as proteases, lipases, a chitinase, and novel proteins (18). T2S is important for both the intracellular infection of host cells and growth in a murine model of Legionnaires’ disease (19–21).

In contrast to T4S effectors, the location of T2S substrates during intracellular infection is unknown. A prevailing view of *L. pneumophila* infection is that the LCV is a tight compartment from which only T4S effectors translocate into the host cell cytosol (22). However, two of our recent observations suggest that T2S substrates might not be restricted to the LCV. First, T2S mutants are impaired in their ability to retain Rab1B on the cytoplasmic face of the LCV, suggesting that a T2S substrate might exit the LCV and engage cytosolic host GTPases (21). Second, the same T2S mutants trigger elevated cytokine levels through the MyD88 and Toll-like receptor 2 signaling pathways in infected human macrophages, suggesting that other T2S substrates might translocate and dampen cytosolic sensors of innate immunity (23, 24). Here, we demonstrate that the ProA and ChiA substrates do, in fact, translocate into the macrophage cytosol and associate with the cytoplasmic face of the LCVM. Additional data suggest that this process occurs in two steps where the substrates are first delivered into the lumen of the LCV via the T2S system and then access the macrophage cytosol through a semipermeable vacuolar membrane.

## RESULTS

### ProA is present at the periphery of the LCV.

**FIG 1:**
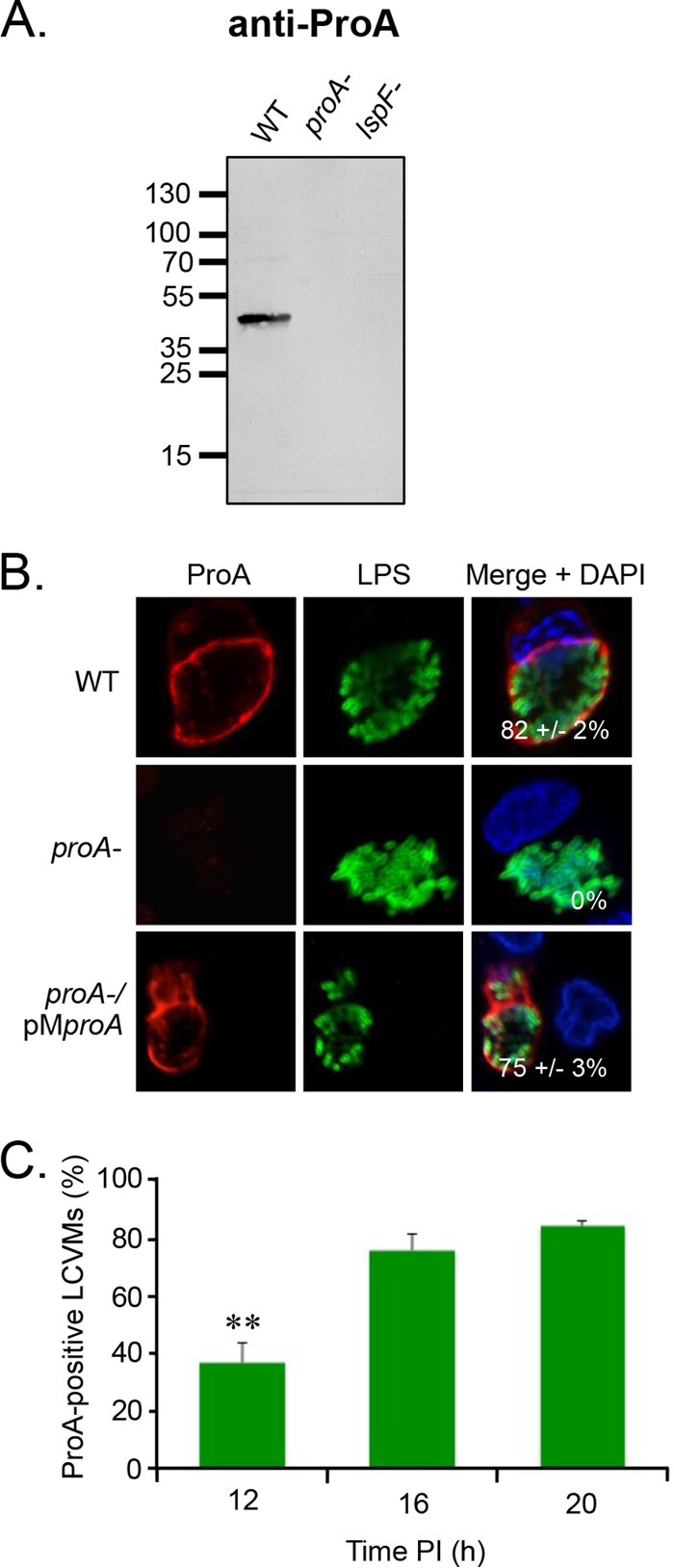
Location of ProA in *L. pneumophila*-infected macrophages. (A) WT strain 130b (WT), *proA* mutant AA200 (*proA*-), and *lspF* mutant NU275 (*lspF*-) were grown in BYE broth, and then culture supernatants were subjected to SDS-PAGE and immunoblot analysis using ProA antiserum. The values corresponding to migration of molecular mass markers (shown in kilodaltons) appear on the left. (B) Human U937 cells were infected with the WT strain, *proA* mutant, or complemented *proA* mutant (*proA*-/pM*proA*) for 16 h and were then analyzed by confocal microscopy using antibodies against ProA (left) and LPS (middle). Host nuclei and bacterial DNA were stained with DAPI (blue), and merged images appear in the right column with the percentages of ProA-positive LCVMs (± standard deviations [SD]) in the lower-right-hand corner. Results presented show a portion of the cell containing the LCV and are representative of two (A) or three (B and C) independent experiments. (C) Quantification of percent ProA localization (± SD) to the LCVM over time, based on the combined results from three independent experiments. Asterisks indicate significant differences in the extent of localization between 12 and 16 h (Student’s *t* test; **, *P* < 0.01). PI, postinoculation.

To begin to analyze the localization of T2S substrates during intracellular infection, we examined ProA, as it is the most abundantly expressed T2S substrate, at least in broth culture (17). ProA is a 38-kDa zinc metalloprotease that mediates damage in animal models of pneumonia (25–27) and has cytotoxic activity against a variety of tissue culture cells (28, 29). Although ProA is not required for replication in human macrophages and *Acanthamoeba castellanii*, it is necessary for optimal replication in *Hartmannella vermiformis* and *Naegleria lovaniensis* (30–32). Polyclonal ProA antiserum was produced in rabbits immunized with purified recombinant FLAG-tagged ProA. To verify the specificity of the antibody, *L. pneumophila* wild-type (WT) strain 130b, *proA* mutant, and T2S (*lspF*) mutant culture supernatants were analyzed by immunoblotting. A band of the expected size was detected in the WT supernatant but was absent in the supernatants of the *proA* and *lspF* mutants (Fig. 1A). To determine the localization of ProA in infected human macrophages, differentiated U937 macrophage-like cells were synchronously infected with strain 130b for 16 h, fixed, permeabilized with Triton X-100 (TX-100), and processed for indirect immunofluorescence analysis (IFA) of ProA by confocal microscopy. Anti-lipopolysaccharide (anti-LPS) monoclonal antibody (MAb) 3/1 was used to delineate bacteria within the LCV. As previously reported, this antiserum detects a phase-variable epitope of LPS that is expressed by all bacteria early in intracellular infection and predominantly by bacteria at the periphery of the vacuole at 15 h or more postinoculation (p.i.) (33). Strikingly, ProA formed a robust ring-like pattern at the periphery of the vacuole in 82% of the infected macrophages (Fig. 1B). This ring was absent in cells infected with the *proA* mutant but was restored in macrophages infected with the complemented *proA* mutant (Fig. 1B), confirming that the observed antiserum labeling is entirely due to ProA. This ring pattern was also seen when WT bacteria were delineated by green fluorescent protein (GFP) expression rather than by the use of anti-LPS MAb 3/1 (see Fig. S1A in the supplemental material). To further analyze the timing of ProA localization, U937 cells were infected for 12, 16, 20, and 24 h and processed for IFA and confocal microscopy. ProA localized to the LCVM in 37% of cells by 12 h, and the proportion increased to ~80% of cells by 16 to 20 h p.i. (Fig. 1C). Lysis of the host cell was observed at 24 h, as previously recorded (11, 21).

10.1128/mBio.00870-17.1 FIG S1  ProA localization in macrophages infected with GFP-expressing bacteria. U937 cells were infected with WT strain 130b (A) or *lspF* mutant NU275 expressing GFP (B) and were then analyzed by confocal microscopy with ProA antiserum. Host nuclei and bacterial DNAs were stained with DAPI. ProA and GFP expression data appear in the left and center columns, respectively, and the merged images appear in the right columns with the percentages of ProA-positive LCVMs (± SD) in the lower-right-hand corner. The images presented show a portion of the cell containing the LCV and are representative of three independent experiments. Download FIG S1, TIF file, 0.2 MB. Copyright © 2017 Truchan et al. 2017 Truchan et al. This content is distributed under the terms of the Creative Commons Attribution 4.0 International license.

### ProA localizes to the cytoplasmic face of the LCVM.

**FIG 2:**
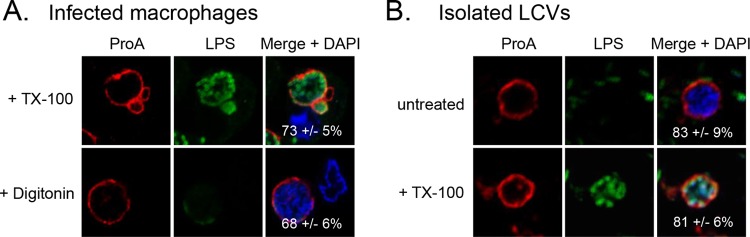
Location of ProA on the LCVM. WT 130b-infected U937 cells permeabilized with either TX-100 or digitonin (A) and LCVs isolated from WT-infected U937 cells that were left untreated or permeabilized with TX-100 (B) were labeled with ProA (left) and LPS (middle) antisera and then analyzed by confocal microscopy. Host nuclei and bacterial DNA were stained with DAPI, and merged images appear in the right column with the percentages of ProA-positive LCVMs (± SD) in the lower-right-hand corner. Results in panel A show a portion of the cell containing the LCV. Data presented are representative of three independent experiments.

To determine if ProA is present on the lumenal or cytoplasmic face of the LCV membrane (LCVM), U937s cells infected with WT *L. pneumophila* were permeabilized with digitonin. Unlike TX-100, which permeabilizes all host and bacterial membranes (34), digitonin selectively permeabilizes the plasma membrane, allowing antibody delivery into the macrophage cytosol only (35). Thus, in contrast to what was seen with TX-100-treated cells (Fig. 1B), the LPS antibody did not label digitonin-treated cells (Fig. 2A). More importantly, the robust ring of ProA was still detected after digitonin treatment. These data suggest that ProA is on the cytoplasmic face of the LCVM. To confirm these results with a method that does not rely on membrane permeabilization, LCVs were liberated from infected U937 cells by Dounce homogenization (36) and were then processed for IFA and confocal microscopy. As shown by analysis of free LCVs, the antibody has direct access to ProA only if it is on the surface of the vacuole. Importantly, the robust ring of ProA was detected similarly on both unpermeabilized and permeabilized free LCVs (Fig. 2B). LPS was detected only in permeabilized LCVs and on surrounding free bacteria (Fig. 2B), confirming the membrane integrity of the isolated vacuoles. We conclude from these data that ProA is present to a great extent on the cytoplasmic face of the LCVM in infected U937 cells.

### ProA localizes to the LCVM as early as 6 h postinoculation.

**FIG 3:**
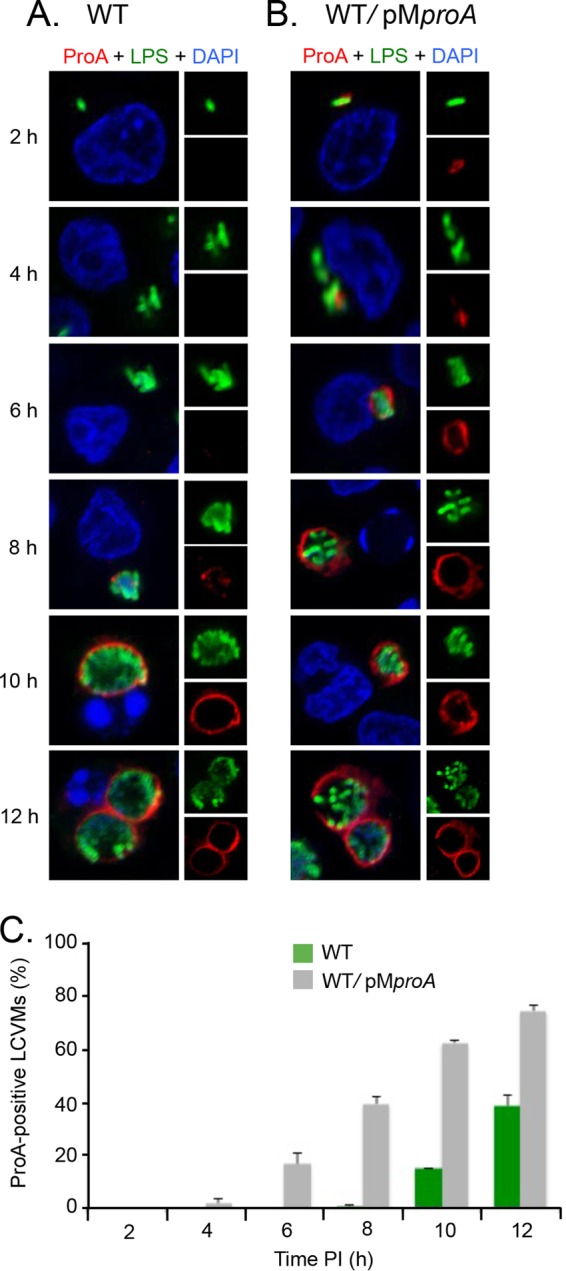
Timing of ProA localization to the LCVM. (A and B) U937 cells were infected for 2, 4, 6, 8, 10, and 12 h with either WT 130b (A) or the WT carrying pM*proA* (B) and were then labeled with LPS antisera (upper-right boxes) and ProA antisera (lower-right boxes) and analyzed by confocal microscopy. Host and bacterial DNAs were stained with DAPI, and the merged images appear as the larger boxes on the left side. Results presented here show a portion of the cell containing the LCV. (C) Quantification of percent ProA localization (± SD) to the LCVM over time, based on combined results from three independent trials.

To determine when during infection ProA first translocates and localizes to the LCVM, U937 cells were synchronously infected with WT 130b and surveyed with ProA and LPS antisera at 2, 4, 6, 8, 10, and 12 h p.i. by confocal microscopy. Expression of ProA was observed starting at 8 h p.i., ~4 h after the start of bacterial replication (Fig. 3A and C). At this time point, ProA could be seen within the lumen of the LCV and was first detected, albeit weakly, at the LCVM. By 10 h p.i., ProA was clearly localized to the LCVM in 15% of infected cells, and the proportion rose to almost 40% by 12 h p.i. (Fig. 3A and C), as had been seen in earlier experiments (Fig. 1C). Interestingly, beyond 8 h, ProA was rarely detected in the LCV, suggesting that ProA translocates very soon after it is expressed and that the timing of translocation is dictated by the timing of ProA expression. To investigate this possibility, U937 cells were infected with the WT strain, which hyperexpresses ProA from the pM*proA* multicopy plasmid, for 2, 4, 6, 8, 10, and 12 h and were examined by confocal microscopy. Expression of ProA was then evident by 2 h p.i., and translocation and localization to the LCVM had clearly occurred by 6 h p.i., 4 h earlier than had been seen with the WT strain (Fig. 3B and C). ProA localized to 40% of LCVMs by 8 h p.i. in cells infected with pM*proA-*containing *L. pneumophila* in contrast to the 12-h time point observed in WT *L. pneumophila* infections (Fig. 3). Together, these results indicate that the timing of ProA translocation is primarily controlled by the timing and level of ProA expression. They also indicate that all other factors, whether host or bacterial, needed for translocation are in place by 6 h p.i.

### ProA translocation and localization to the LCVM are conserved in human macrophages and amoebae and with other strains of *L. pneumophila*.

**FIG 4:**
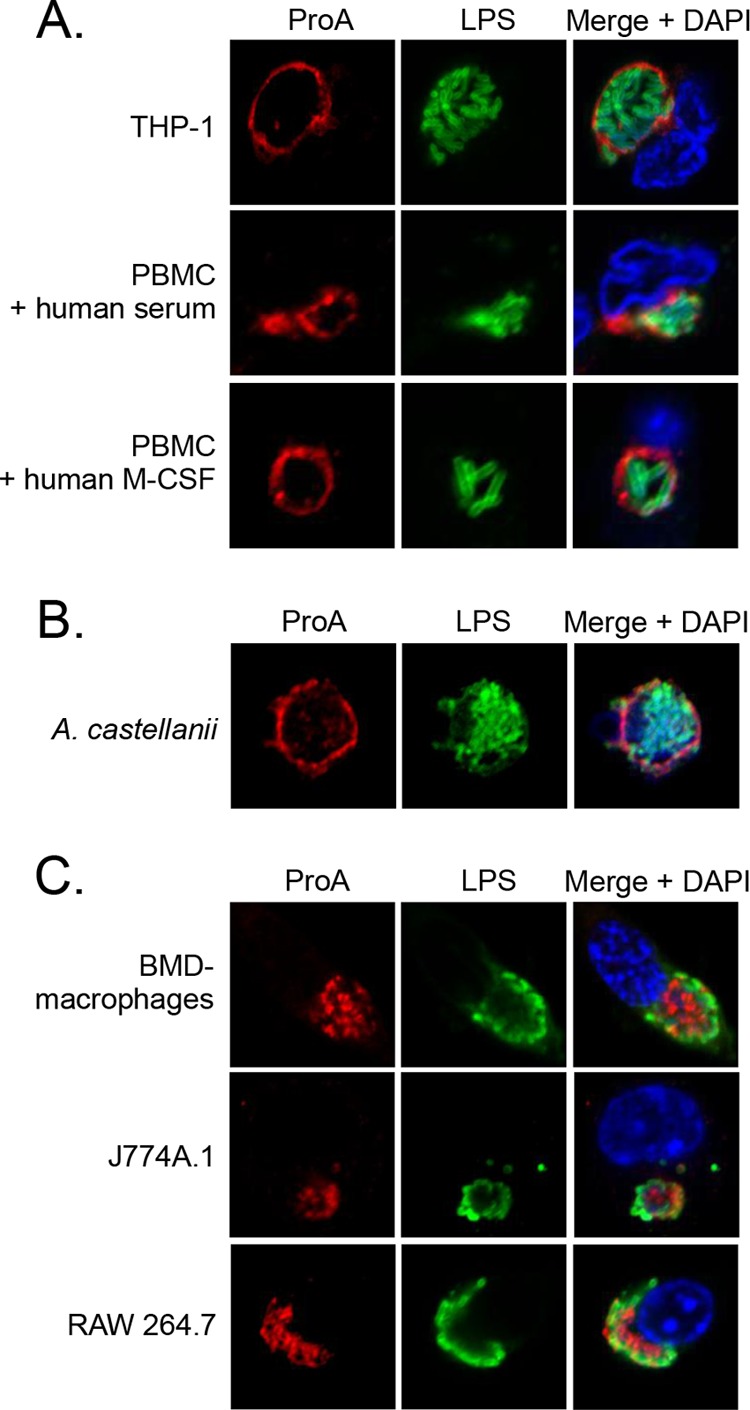
ProA translocation within different infected host cells. (A) Human THP-1 cells and PBMCs differentiated with human serum or human recombinant M-CSF. (B) *A. castellanii* amoebae. (C) BMD macrophages from A/J mice, J774A.1 cells, and RAW 264.7 cells were infected with WT 130b and then analyzed by confocal microscopy using ProA (left) and LPS (center) antisera. Host and bacterial DNA were stained with DAPI, and merged images appear in the rightmost columns. The images presented show a portion of the cell containing the LCV and are representative of two (PBMC + human M-CSF; panel A) or three independent experiments.

**FIG 5:**
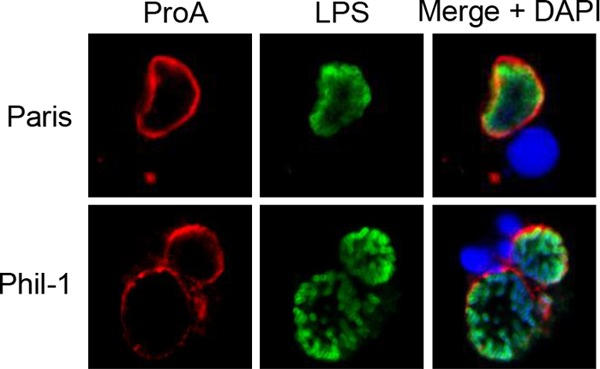
ProA translocation by different *L. pneumophila* strains. U937 cells were infected with WT strains Paris and Philadelphia-1 (Phil-1) and were then analyzed by confocal microscopy with ProA and LPS antisera. Host nuclei and bacterial DNAs were stained with DAPI. The results presented show a portion of the cell containing the LCV and are representative of three independent experiments.

To examine if ProA translocation is conserved and not just a peculiarity of U937 cells, localization in a variety of other host cell types was examined. First, we examined the human THP-1 macrophage cell line and human peripheral blood mononuclear cells (PBMCs). We utilized PBMCs differentiated either in human serum or in the presence of human macrophage colony-stimulating factor (M-CSF), as previously described (37, 38). Like U937 cells, these cells have been widely used to study the cell biology of *L. pneumophila* infection (23, 39–41). In all cells, ProA exhibited a ring at the LCVM (Fig. 4A). We next detected this pattern of ProA localization in *A. castellanii*, the major environmental amoebal host for *L. pneumophila* (Fig. 4B) (42). Finally, we assessed ProA localization in mouse macrophages. We utilized bone marrow-derived (BMD) macrophages obtained from A/J mice, as well as RAW 264.7 and J774A.1 macrophage cell lines, since these cell types are used in the *Legionella* field (21, 43–45). Curiously, ProA localization to the LCVM was absent in all of the mouse macrophages infected with WT *L. pneumophila* (Fig. 4C). Given that most primary mouse-derived macrophages are resistant to *L. pneumophila* infection as a result of flagellin activation of the mouse-specific variant of Nod-like receptor Naip5 (46, 47), we wondered if the lack of translocation that we observed in murine macrophages could be attributed to the activation of Naip5. However, translocation of ProA was still not observed when we infected various murine BMD macrophages with a flagellin mutant (Fig. S2). Taken together, these data suggest that there might be differences in translocation into the cytosol in human versus murine macrophages. To determine if other strains of *L. pneumophila* also translocate ProA, U937 cells were infected with strains Philadelphia-1 (Phil-1) and Paris. Like 130b, these strains are clinical isolates that have been extensively utilized in the analysis of *L. pneumophila* infection (48). ProA was seen in a ring-like pattern surrounding the LCVs of both strains (Fig. 5). Together, these results indicate that ProA translocation is not just an anomaly of infection with strain 130b and is likely common during infection by different strains and within different human and amoebal host cell types. This conservation suggests that the association of ProA with the cytoplasmic face of the LCVM has a functional role in intracellular infection.

10.1128/mBio.00870-17.2 FIG S2  Localization of ProA in BMD macrophages from C57BL/6 and A/J mice infected with a flagellin mutant. BMD macrophages from C57BL/6 and A/J mice were infected with *fla* mutant NU347 and then analyzed by confocal microscopy with ProA and LPS antisera. Host nuclei and bacterial DNAs were stained with DAPI. The images presented show a portion of the cell containing the LCV and are representative of two independent experiments. Download FIG S2, TIF file, 0.2 MB. Copyright © 2017 Truchan et al. 2017 Truchan et al. This content is distributed under the terms of the Creative Commons Attribution 4.0 International license.

### ProA translocation into the macrophage cytosol is dependent on type II secretion.

**FIG 6:**
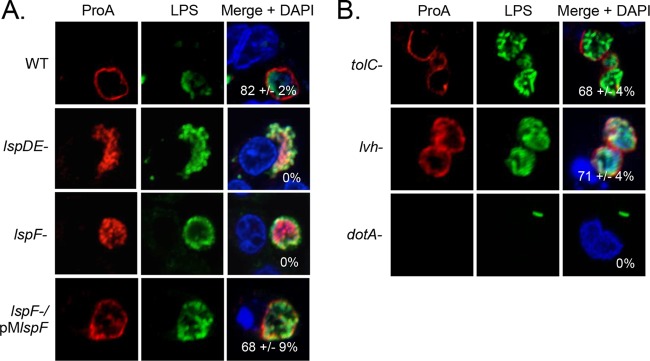
Effect of the T2S, T1S, and T4S on ProA translocation. U937 cells were infected with WT strain 130b, *lspDE* mutant NU258 (*lspDE-*), *lspF* mutant NU275 (*lspF*-), and a complemented *lspF* mutant (*lspF*-/pM*lspF*) (A) and *tolC* mutant NU390 (*tolC*-), *lvh* mutant AA474, and *dotA* mutant GG105 (B) and were then analyzed by confocal microscopy using ProA (left) and LPS (center) antisera. Host nuclei and bacterial DNAs were stained with DAPI, and merged images appear in the right columns with the percentages of ProA-positive LCVMs (± SD) in the lower-right-hand corner. The results presented show a portion of the cell containing the LCV and are representative of three independent experiments.

To date, the only *L. pneumophila* proteins reported to be trafficked into the cytosol of infected human or amoebal cells are the effectors of the Dot/Icm type IVB secretion (T4BS) system (13, 49). Given this, we next determined if ProA localization to the LCVM is, in fact, dependent on the T2S. U937 cells were infected with an *lspDE* mutant and the *lspF* mutant and were examined by confocal microscopy. Strikingly, in the T2S mutant-infected cells, ProA no longer localized to the LCVM and was completely contained within the LCV (Fig. 6A). This result was also observed using GFP-expressing mutant bacteria, where the ProA signal completely overlapped the GFP signal (Fig. S1B). When the *lspF* mutant was complemented, ProA localization to the LCVM was restored (Fig. 6A). Thus, ProA localization to the LCVM is dependent on the T2S.

In addition to the T2S and the Dot/Icm T4BS system, *L. pneumophila* encodes two functional secretion systems—type 1 secretion (T1S) and Lvh type 4A secretion (T4AS). To first examine if ProA translocation is also dependent on T1S in addition to T2S, U937 cells were infected with a *tolC* mutant of strain 130b, as *tolC* encodes the outer membrane component of the secretion apparatus (50). The ring of ProA was still observed in this mutant (Fig. 6B), indicating that T1S is not involved. To determine if translocation is dependent on T4AS, we studied a mutant where the entire *lvh* locus was deleted (51). This mutant also showed a robust ring of ProA at the LCVM (Fig. 6B). Finally, to try to determine if translocation is dependent on the Dot/Icm type 4B system, we utilized a *dotA* mutant lacking the type 4 secretion system inner membrane DotA protein (52). However, the *dotA* mutant was completely unable to replicate within the macrophages (Fig. 6B), as has been previously reported (53, 54), and thus, we were not able to assess expression of ProA. Nonetheless, we do not believe that ProA is a Dot/Icm substrate, since multiple broad genetic and bioinformatic screens have led to the identification of >320 T4S effectors but none identified ProA (14, 15, 55–58). Given that finding and our data on the T2S, T1S, and T4AS mutants, we hypothesize that translocation occurs in two steps: (i) ProA is trafficked into the vacuolar lumen by the T2S system, and (ii) ProA traffics across the LCVM and into the macrophage cytosol via a novel mechanism.

### The type II secretion substrate ChiA also localizes to the cytoplasmic face of the LCVM.

**FIG 7:**
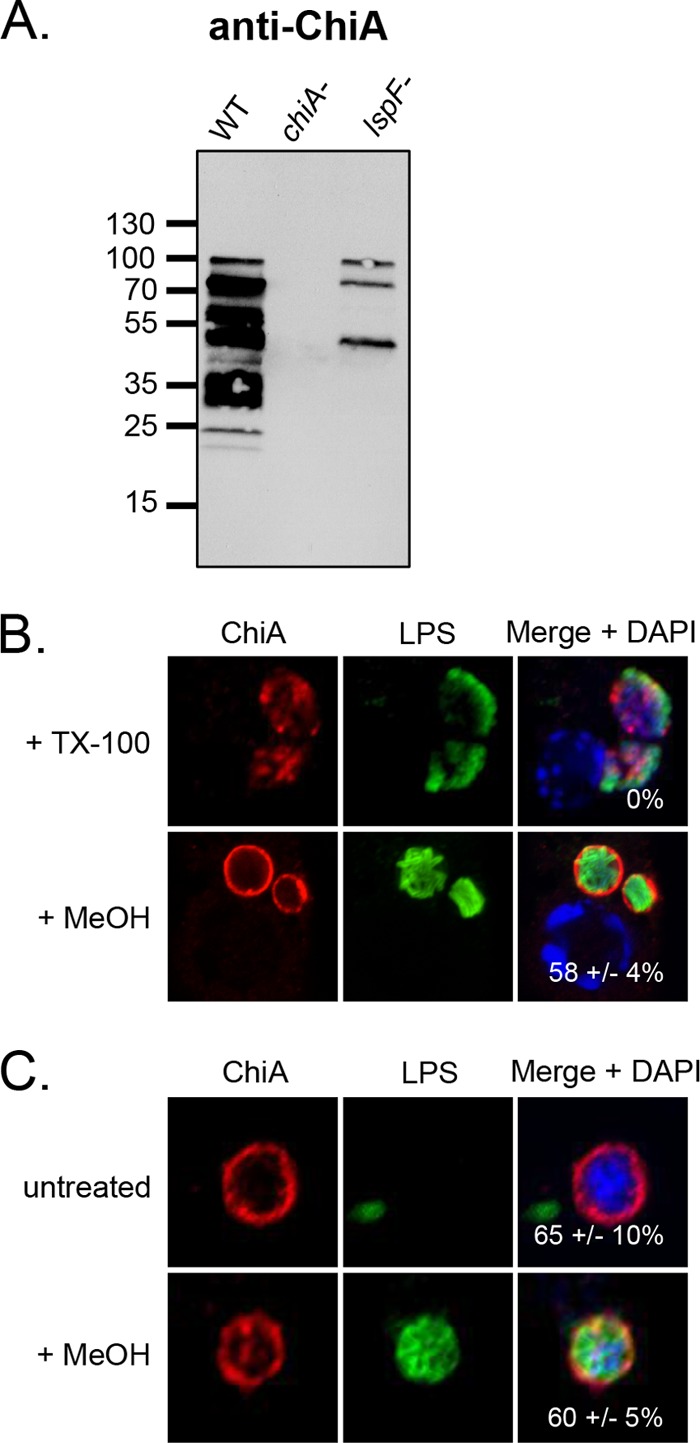
Location of ChiA in *L. pneumophila-*infected macrophages. (A) WT 130b, *chiA* mutant NU318 (*chiA*-), and *lspF* mutant NU275 (*lspF*-) were grown in BYE broth, and culture supernatants were then subjected to SDS-PAGE and immunoblot analysis with ChiA antiserum. (B) U937 cells were infected with WT 130b and permeabilized with TX-100 or MeOH and were then analyzed by confocal microscopy with ChiA (left column) and LPS (center column) antisera. The images presented show a portion of the cell containing the LCV. (C) LCVs obtained from WT 130b-infected U937 cells were labeled with ChiA and LPS antisera and then analyzed by confocal microscopy. (B and C) Host nuclei and bacterial DNAs were stained with DAPI, and merged images appear in the right columns with the percentages of ChiA-positive LCVMs (± SD) in the lower-right-hand corner. The data presented are representative of three independent experiments.

To begin to determine if other T2S substrates traffic into the macrophage cytosol, we generated antisera against ChiA. *L. pneumophila* ChiA is an 81-kDa enzyme that degrades chitin, an insoluble polymer found in the cell walls of mold, fungi, and algae (59). Interestingly, a *chiA* mutant of *L. pneumophila* showed a reduced ability to persist in the lungs of infected mice (59). Confirming its specificity, the ChiA antiserum recognized a band of the appropriate molecular weight in the WT supernatant but did not recognize a band in the *chiA* mutant supernatant (Fig. 7A). That ChiA exhibited a multibanding pattern suggests that it is modified or degraded during culture. Recognition of ChiA was visible but noticeably diminished in the *lspF* mutant supernatant, confirming the importance of the T2S system in exporting this protein. The small amount that was detected was likely due to some lysis of the bacteria and/or to the presence of the substrate in outer membrane vesicles (OMVs) (60). To examine the localization of ChiA in infected host cells, U937 cells were infected with WT 130b for 16 h and processed for IFA with LPS and ChiA antisera. When the cells were permeabilized with TX-100, as had been done for ProA, the ChiA signal was observed in the vacuole and overlapped the bacteria (Fig. 7B). However, when the cells were permeabilized with methanol (MeOH), which, unlike TX-100, permeabilizes host membranes but does not permeabilize the peptidoglycan layer of *L. pneumophila* (61), ChiA gave a ring-like pattern surrounding the LCV in 58% of infected cells. To test whether ChiA is present on the cytoplasmic or lumenal face of the LCVM, we analyzed free LCVs. The ChiA antiserum labeled the unpermeabilized and permeabilized LCVs similarly (Fig. 7C), indicating that ChiA, like ProA, is present on the cytoplasmic face of the vacuole. In light of our ChiA result, we next examined T2S substrate CelA, a cellulase, for its localization during infection after TX-100 and MeOH permeabilization. We were not able to observe CelA associated with the LCVM at 16 h p.i. (Fig. S3), implying that not all T2S substrates translocate across the LCVM. However, it is possible that CelA translocates into the macrophage cytosol but does not localize to the LCVM and is too diffused for detection.

10.1128/mBio.00870-17.3 FIG S3  Localization of CelA in *L. pneumophila-*infected macrophages. (A) The WT strain, *celA* mutant NU353 (*celA*-), and *lspF* mutant NU275 (*lspF*-) were grown in BYE broth, and culture supernatants were then subjected to SDS-PAGE and immunoblot analysis performed with CelA antiserum. (B) U937 cells were infected with WT strain 130b, permeabilized with TX-100 or MeOH, and analyzed by confocal microscopy with CelA (left) and LPS (center) antisera. Host nuclei and bacterial DNAs were stained with DAPI, and the merged images appear in the right column. The images presented show a portion of the cell containing the LCV (B) and are representative of two (A) or three (B) independent experiments. Download FIG S3, TIF file, 0.4 MB. Copyright © 2017 Truchan et al. 2017 Truchan et al. This content is distributed under the terms of the Creative Commons Attribution 4.0 International license.

**FIG 8:**
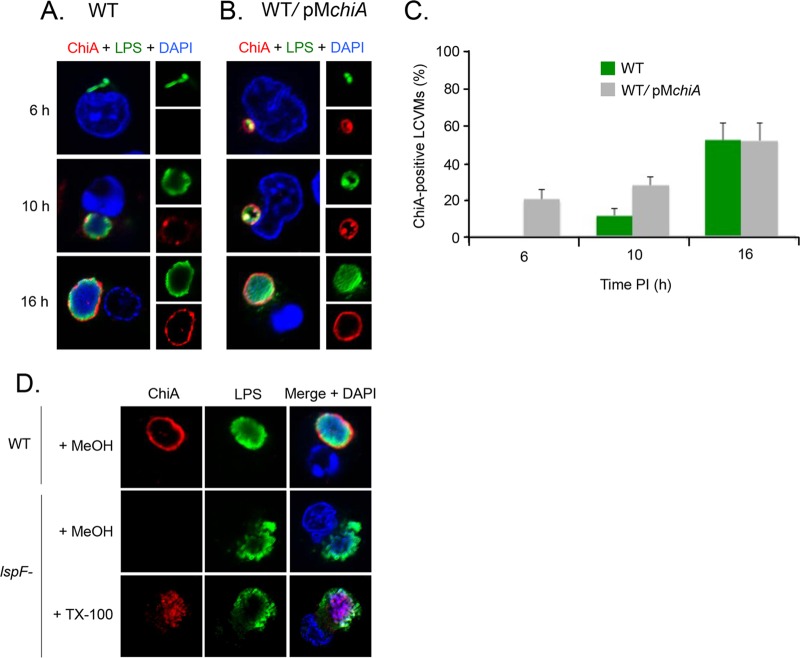
Timing and T2S dependency of ChiA translocation. U937 cells were infected for 6, 10, and 16 h with either WT 130b (A) or the WT carrying pM*chiA* (B) and were then labeled with LPS (upper-right boxes) and ChiA (lower-right boxes) antisera and analyzed by confocal microscopy. Host and bacterial DNAs were stained with DAPI, and the merged images appear as the larger boxes on the left side. (C) Quantification of percent ChiA localization (± SD) to the LCVM over time, based on the combined results from three independent trials. (D) U937 cells were infected with WT 130b and *lspF* mutant NU275, permeabilized with TX-100 or MeOH, and analyzed by confocal microscopy with DAPI and using ChiA and LPS antisera. The images presented show a portion of the cell containing the LCV and are representative of three independent experiments.

To determine when ChiA is first capable of localizing to the LCVM, U937 cells were infected with WT *L. pneumophila* or the WT strain hyperexpressing ChiA from an inducible plasmid (pM*chiA*) and were analyzed at 6, 10, and 16 h p.i. Overexpressed ChiA clearly localized to the LCVM as early as 6 h p.i. (Fig. 8B). In contrast, ChiA from WT did not associate with the LCVM until 10 h p.i., as had been observed for ProA, but the intensity of the ring increased and was comparable to that of the ProA ring at the 16-h time point (Fig. 8A to C). This timing overlaps the localization of ProA and further suggests that all factors needed for the translocation of T2S substrates are in place by 6 h p.i. To next determine if ChiA translocation is also dependent on T2S, the *lspF* mutant was tested. Similar to ProA, ChiA was contained in the vacuole and, as visualized after permeabilization with TX-100, was completely contained within the bacteria (Fig. 8D). Together, these data indicate that ChiA also localizes to the LCVM as early as 6 h p.i. in a T2S-dependent manner.

### Galectin localizes within the LCV of human macrophages as early as 6 h postinoculation.

**FIG 9:**
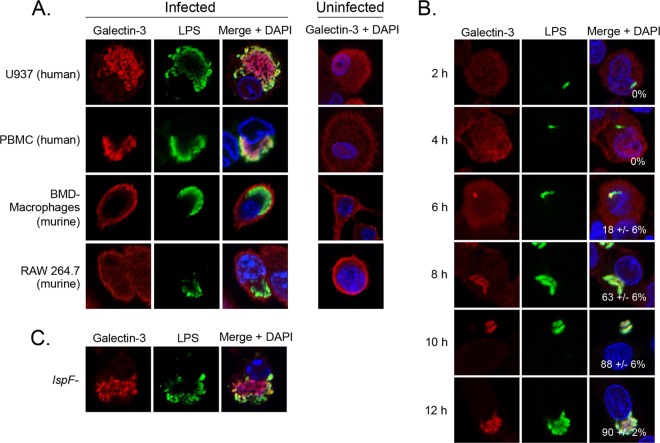
Galectin localization in *L. pneumophila*-infected macrophages. (A) U937 cells, PBMCs differentiated with human serum, BMD macrophages from A/J mice, and RAW 264.7 cells were infected with WT 130b for 16 h or were left uninfected (rightmost column) and were analyzed by confocal microscopy with galectin-3 (Santa Cruz Biotechnology) (left) and LPS (center) antisera. Host and bacterial DNAs were stained with DAPI, and merged images appear in the right column. (B and C) U937 cells were infected with WT 130b for 2, 4, 6, 8, 10, and 12 h (B) or with the *lspF* mutant NU275 for 16 h (C) and were then analyzed by confocal microscopy with galectin-3 (Santa Cruz Biotechnology) and LPS antisera. Host and bacterial DNAs were stained with DAPI, and merged images appear in the right columns with the percentages of galectin-positive LCVMs (± SD) in the lower-right-hand corner. The images presented are representative of three independent trials.

We envisioned that the second step of translocation after secretion of the T2S substrates into the vacuolar lumen might involve a permeable LCVM. To assess this, we analyzed galectin-3, a eukaryotic protein whose localization changes in response to the presence of damaged host membranes. Indeed, galectin-3 has been widely utilized to examine vacuolar integrity for other intracellular pathogens, such as *Salmonella enterica* serovar Typhimurium, *Shigella flexneri*, and *Trypanosoma cruzi* (62, 63). To begin, we infected a variety of macrophages with WT 130b for 16 h and examined galectin-3 localization by IFA. Strikingly, galectin-3 clearly localized within the LCVs of U937 cells and differentiated PBMCs and associated with the bacteria (Fig. 9A). In uninfected cells, the galectin-3 antibody gave a diffused cytosolic labeling (Fig. 9A). That galectin-3 associated with the bacteria within the LCV is compatible with the observation that galectin-3 can bind to the surface of bacteria, including *Mycobacterium tuberculosis* and *Pseudomonas aeruginosa* (64–66). Localization within the LCV was also observed when we employed a second galectin-3 antibody obtained from an alternative source, although the degree of association with the bacteria was less pronounced (Fig. S4). Interestingly, galectin-3 did not localize in the LCV of infected murine macrophages (Fig. 9A), just as ProA translocation was not evident in this cell type (Fig. 4). Indeed, the galectin-3 assay results appeared similar in infected and uninfected cells. To further examine this, infected U937 cells were labeled with an antibody against galectin-8, which also localizes to damaged vesicles but has not been shown to bind to the surface of bacteria (67). Galectin-8 was also observed within the LCV, albeit exhibiting a more punctate pattern that did not colocalize with the bacteria (Fig. S4).

10.1128/mBio.00870-17.4 FIG S4  Localization of galectin-3 and galectin-8 in *L. pneumophila-*infected macrophages. U937 cells were infected with WT 130b for 16 h or were left uninfected and were analyzed by confocal microscopy with galectin-3 (Abcam, Inc.), galectin-8, and LPS antisera. Host nuclei and bacterial DNAs were stained with DAPI. The images presented show a portion of the cell containing the LCV and are representative of three independent experiments. Download FIG S4, TIF file, 0.3 MB. Copyright © 2017 Truchan et al. 2017 Truchan et al. This content is distributed under the terms of the Creative Commons Attribution 4.0 International license.

To assess when the LCVM is first permeable to galectin-3, U937 cells were analyzed at 2, 4, 6, 8, 10, and 12 h p.i. Remarkably, galectin-3 localized within the LCV as early as 6 h p.i., the same time point where we were first able to visualize ProA and ChiA at the LCVM (Fig. 9B). These data suggest that the LCV is permeable as early as 6 h p.i. and that ProA and ChiA might exit the LCV through a permeable membrane. That permeability is detected as early as 6 h p.i. suggests that this is neither a late-stage effect of extensive intracellular replication nor a prelude to host cell lysis. As the T2S system delivers many degradative enzymes, including lipolytic enzymes (68), we next determined if the presence of the permeable membrane is dependent on the T2S system. U937 cells were infected with the *lspF* mutant for 16 h and galectin-3 localization was analyzed. Galectin-3 localized within the LCV (Fig. 9C) and did so similarly to the manner seen with infection with WT *L. pneumophila*, indicating that the T2S system is not required for the permeable LCVM.

## DISCUSSION

Here, we demonstrate that the T2S substrates ProA and ChiA translocate out of the LCV and into the macrophage cytosol, where they appear in a ring-like pattern around the LCVM. When *L. pneumophila* is grown in broth cultures, ProA and ChiA exist within OMVs, in addition to being released into the extracellular milieu via the T2S system (59, 60). OMVs have also been previously detected in the LCV in infected macrophages (60). However, since *L. pneumophila* mutants lacking T2S do not exhibit ProA and ChiA localization around the LCVM, the translocation event most likely begins with the secretion of “free” protein into the vacuolar lumen via the T2S system, followed by a second trafficking event across the LCVM. Translocation across the LCVM occurred with all clinical isolates tested and was also evident in infected amoebae, heightening the significance of our findings. The predominant view in the *L. pneumophila* field has been that the only protein effectors that gain access to the host cell cytosol during intracellular infection are those of the Dot/Icm T4S system (22). Our data represent a shift in this paradigm. Furthermore, our observation of galectin-3 localization within the LCV in human macrophages, but not within mouse macrophages where ProA did not translocate, suggests that the T2S substrates access the host cytosol through a permeable LCV. Others have examined the permeability of the LCV but have done so primarily using murine BMD macrophages with or without gamma interferon (IFN-γ) treatment and/or at very early infection times (54, 69, 70). In all cases, little to no permeability was detected and the authors therefore did not conclude that *L. pneumophila* exists naturally in a semipermeable vacuole. As such, the LCVM has been thought to be impermeable with respect to the export of proteins, being susceptible only to the action of the T4S apparatus. To our knowledge, the current report presents the first substantial data set that led to a contrasting conclusion.

Given that galectin-3, galectin-8, ProA, and ChiA cross the LCVM at 40, 40, 38, and 80 kDa, respectively, but that the IgG anti-LPS antibodies (at 150 kDa) cannot, we surmise that the vacuolar membrane is only semipermeable and is not grossly damaged or compromised. Although we observed ProA and ChiA at the LCVM from 6 to 20 h, the amount of translocated protein appeared to increase from 8 to 10 h for ProA (see Fig. 3A) and from 10 to 16 h for ChiA (see Fig. 8A). This suggests that the permeability of the LCVM is not transient and occurs for at least 10 h, i.e., between 6 and 16 h postentry while the LCV is growing in size. As noted above, the LCVM is formed from the plasma membrane during phagocytosis and the LCV undergoes fusion with the ER and ER-Golgi intermediate compartment vesicles within the first few hours after bacterial entry (8, 71). Consequently, as early as 4 h p.i., the LCVM contains a variety of plasma membrane and ER membrane proteins (72, 73). Thus, we posit that the semipermeability of the LCVM might be due to the early acquisition of a host-derived membrane transporter(s) which is capable of translocating ProA and ChiA. Interestingly, vacuoles that harbor *Mycobacterium tuberculosis* contain a host membrane transporter derived from the ER that allows the translocation of mycobacterial proteins of up to 70 kDa in size into the macrophage cytosol (74–76). Thus, co-opting of host membrane transporters to deliver effector proteins into the host cytosol may prove to be a common strategy of intracellular parasites. However, an alternative hypothesis to explain the translocation event is that a nonspecific pore is formed in the LCVM when the Dot/Icm T4S apparatus pierces the vacuolar membrane, thereby allowing the “accidental” leakage of non-T4S substrates from the lumen of the LCV into the host cytosol. Such a scenario has been theorized to be responsible for the translocation of *L. pneumophila* flagellin (46). Furthermore, in the case of *Burkholderia cenocepacia*, proteases are secreted into the lumen of the pathogen-occupied vacuole via the T2S system and then access the macrophage cytosol through the vacuolar membrane that has been disrupted by the type VI secretion system (77). As a third explanation for our observations, a *L. pneumophila* factor secreted into the lumen of the LCV might create a pore in the LCVM that allows translocation. Our analysis of a panel of secretion mutants indicates that the T2S, T1S, and Lvh T4S systems are not required for the permeability of the vacuole. However, the Dot/Icm T4S system or a yet-to-be-defined secretion system could be delivering a pore-forming effector into the LCV. Given that the galectin proteins and ProA and ChiA, representing three structurally distinct proteins, are translocated across the LCVM, we surmise that the second step of translocation is relatively nonspecific. Thus, we posit that other T2S substrates of *L. pneumophila* access the cytosol of infected host cells.

Both ProA and ChiA exhibit a striking ring-like pattern on the cytoplasmic face of the LCVM, suggesting that the two proteins form an association with the vacuolar membrane. In the case of ProA, that association may be due to a putative farnesylation domain (-CYVD) at the C terminus of the protein (78, 79). Farnesylation is a type of eukaryotic posttranslational modification that adds an isoprenyl lipid moiety to a C-terminal cysteine residue (78, 80). This moiety can intercalate into the peripheral region of a lipid bilayer and thereby facilitate membrane association. Indeed, the *L. pneumophila* Dot/Icm T4S effector AnkB associates with the LCVM through farnesylation (81). In thinking how ChiA might associate with the LCVM, it could be relevant that ChiA, unlike ProA, was observed at the LCVM following MeOH but not TX-100 permeabilization. MeOH permeabilizes cells by dissolving lipids from membranes, whereas TX-100 can nonselectively extract proteins along with lipids (34, 82). Therefore, the apparent tethering of ChiA to the LCVM could be the result of a protein-protein interaction, analogous to the manner in which the Dot/Icm T4S effector PieA localizes to the LCVM (83). Additional possibilities derive from studies of other Dot/Icm effectors such as SidG, which uses a hydrophobic domain to insert into the LCVM (84), or LidA, SetA, and SidM (DrrA), which bind to phosphatidylinositol 4-phosphate or phosphatidylinositol 3-phosphate in the LCVM (81, 85, 86). Although ProA and ChiA were clearly evident near the cytoplasmic face of the LCV, it is possible that they also spread to other locations within the host cell and that our microscopic analysis was not sensitive enough to detect this.

Because ProA and ChiA gain access to the host cell cytoplasm, they likely have a broader role in intracellular infection than previously imagined. Indeed, by not being restricted to the lumen of the LCV, these T2S substrates, like the myriad Dot/Icm effectors, have the potential to promote bacterial growth and/or alterations in host function. Since ProA and ChiA do not clearly appear on the LCVM until approximately 10 h p.i., the translocation event is probably not required for the genesis of the LCV or the earliest rounds of *L. pneumophila* replication. Rather, we posit that translocated ProA and ChiA are involved in the middle stages of intracellular infection and in further maturation of the LCV, processes that are relatively insufficiently studied in the *Legionella* field. *In vitro*, the ProA metalloprotease degrades a broad range of substrates, including extracellular matrix proteins and cytokines (24, 87). Therefore, there may be many cytosolic proteins that are susceptible to ProA action. However, given that it accumulates at or on the LCVM, ProA is likely acting most significantly upon nearby host and/or bacterial proteins whose cleavage or degradation might be needed for optimal maturation of the LCV. Proteomic analysis has revealed that the makeup of the LCV does change over time, with some proteins, including Dot/Icm effectors, appearing early and then disappearing (73, 88, 89). In the case of ChiA, we hypothesize that the translocated chitinase is capable of cleaving *O-*GlcNAcylated proteins (59, 90), which may be present at or near the LCVM. The vast majority of the Dot/Icm T4S effectors that gain access to the host cell cytoplasm are not absolutely required for bacterial intracellular replication, as the individual effector mutants do not replicate to lower numbers than WT bacteria (91). Rather, many of these proteins have subtler and, in some instances, overlapping functions. Although not necessarily required for optimal replication, ProA and ChiA could be mediating processes that impact downstream events, such as signaling of the innate immune system or destruction of the host cell and bacterial spread.

In sum, we have documented that T2S substrates of *L. pneumophila* translocate out of the LCV and into the host cell cytoplasm, where they accumulate at the LCVM. This translocation event correlated with the appearance of a semipermeable LCVM as early as 6 h p.i. Taken together, these observations represent a shift in our view of the LCV from considering it impermeable, except for the translocation of Dot/Icm T4S effectors, to considering it to represent a compartment that is more open, permitting potentially many other bacterial factors to access the host cell cytoplasm and beyond. This significantly expands the potential ways in which *L. pneumophila* might alter or damage its host. Additionally, the observations indicating that translocation and membrane permeability occur in human but not murine macrophages suggest that there are more differences during intracellular infection of these two hosts than previously appreciated (23).

## MATERIALS AND METHODS

### Bacterial strains, media, and chemicals.

*L. pneumophila* strain 130b, also known as AA100 (American Type Culture Collection [ATCC], strain number BAA-74) is a clinical isolate that served as our principal WT strain (92). *lspF* mutant NU275, *lspDE* mutant NU258, a complemented *lspF* mutant, *proA* mutant AA200, *chiA* mutant NU318, *celA* mutant NU253, *dotA* mutant GG105, *tolC* mutant NU390, *lvh* mutant AA474, and *fla* mutant NU347 have been previously described (20, 51, 54, 59, 93–95). Other WT strains examined were strains Paris and Philadelphia-1 (Phil-1) (48). Plasmids expressing pMGFP, pM*proA*, and pM*chiA* have been previously described (21, 31, 59). Legionellae were routinely cultured at 37°C on buffered charcoal yeast extract (BCYE) agar or in buffered yeast extract (BYE) broth which, when appropriate, was supplemented with antibiotics (21). *Escherichia coli* strains DH5α and BL21(DE3) were cultured on LB agar and in LB broth for cloning experiments and in Terrific Broth (TB) for assessments of protein expression. Chemicals were from Sigma-Aldrich unless otherwise noted.

### Recombinant protein and antiserum production.

Genes encoding *celA*, *chiA*, and *proA* were PCR amplified using 5′ GGACAGGGTCTCTCATGAAAATATTTAAGTTTAGCAGTTG 3′ and 5′ ATAATACTCGAGATTAAAATAAGGCTTCAATGTTTG 3′ for *celA*, 5′ GGACAGGGTCTCTCATGCGATATTTATTATTACTGCC 3′ and 5′ ATAATACTCGAGCTCACAAACACCATTAATAGC 3′ for *chiA*, and 5′ GGACAGGGTCTCTCATGCACCCAAATTATTATTT 3′ and 5′ ATAATACTCGAGATCGACATAACAAGATTGAT 3′ for *proA* and cloned into pET28a (Novagen, EMD Millipore). BL21(DE3) colonies containing the recombinant expression plasmid were resuspended in 5 ml TB and used to inoculate 500 ml TB with kanamycin in a 2-liter flask to an initial optical density at 600 nm (OD_600_) of 0.1. Flasks were incubated at 37°C with shaking at 250 rpm until an OD_600_ of 0.7 was reached. Isopropyl-β-d-1-thiogalactopyranoside (IPTG) was added to reach a final concentration of 1 mM for induction of protein expression, and the cell suspension was incubated for an additional 4 h. Cells were harvested by centrifugation at 6,000 × *g* for 10 min, and the pellet was frozen at −20°C until ready for processing. Bacterial pellets were thawed at room temperature, resuspended in 5 ml of Extraction buffer (20 mM Tris-Cl [pH 7.9], 0.5 M NaCl, 10% glycerol, 30 mM imidazole) with cOmplete protease inhibitor cocktail (Roche Life Science) and lysozyme at 0.1 mg/ml and were incubated for 30 min on ice. Cell suspensions were sonicated for 8 cycles (15 s on and 30 s off) at 30% power using the microtip of a Branson sonicator (Branson). Unbroken cells and cell debris were removed by centrifugation at 27,000 × *g* for 15 min at 4°C. Supernatants containing extracted protein were passed through a 0.2-μm-pore-size syringe filter before loading onto an affinity column. Ni^++^ affinity columns for gravity flow chromatography were prepared by adding 2 ml 50% Ni^++^ slurry (Qiagen) to disposable liquid chromatography columns (Thermo Fisher Scientific) and were allowed to settle for a 1-ml bed volume. Columns were first washed with 5 column volumes (CV) of double-distilled water (ddH_2_O) and equilibrated with 5 CV Extraction buffer. Cell lysate was loaded onto the equilibrated Ni^++^ affinity columns at a low flow rate. The columns were washed with 10 CV Extraction buffer, and protein was eluted at a low flow rate using Extraction buffer containing 250 mM imidazole. Fractions (1 ml) were collected and analyzed for protein purity by SDS-PAGE. Fractions with 90% or greater purity were pooled and concentrated, and the Extraction buffer was exchanged with phosphate-buffered saline (PBS). Purified recombinant proteins were submitted to Lampire Biological Laboratories (Pipersville, PA) at a concentration of 2 mg/ml for production of rabbit polyclonal antisera.

### Immunoblot analyses of secreted proteins.

*L. pneumophila* strains were grown to an OD_660_ of 1.8 to 2.0 in a shaking incubator at 230 rpm at 37°C. Supernatants were isolated by centrifugation of the cultures at 5,000 × *g* at 4°C, followed by filtration through a 0.2-μm-pore-size membrane (EMD Millipore). Supernatant protein was concentrated 25× (vol/vol) as follows. Isopropanol (100 ml) was added to 50 ml of culture supernatant and incubated at −20°C overnight. Precipitated protein was centrifuged at 10,000 × *g* for 30 min at 4°C, and the resulting pellet was washed twice in 70% ethanol and resuspended in 2 ml of PBS with added cOmplete protease inhibitor cocktail. Sample volumes were normalized to the measured bacterial optical density, diluted in SDS-loading buffer, and analyzed by immunoblotting as previously described (21). Primary antisera were used at the following concentrations: CelA, 1:5,000; ChiA, 1:10,000; and ProA, 1:5,000 (in 1% milk [wt/vol]–Tris-buffered saline [TBS-T]). The secondary antibody, goat anti-rabbit horseradish peroxidase antibody (Cell Signaling Technology, Inc.), was diluted 1:10,000 in 1% milk–TBS-T.

### Cultivation, differentiation, and infection of host cell lines.

Human U937 (ATCC CRL-1593.2), THP-1 (ATCC TIB 202), RAW 264.7 (ATCC TIB-71), and J774A.1 (ATCC TIB-67) cell lines were maintained in RPMI 1640 medium (Gibco) supplemented with 10% fetal bovine serum (FBS) (RPMI FBS) at 37°C in a humidified incubator with 5% CO_2_. U937 cells were differentiated 72 h prior to infection in RPMI FBS with 20 ng/ml phorbol 12-myristate 13-acetate (PMA) (96). THP-1 cells were differentiated with 100 ng/ml PMA 16 to 20 h prior to infection (23). The amoeba *A. castellanii* (ATCC 30234) was grown and maintained at 35°C in 712 PYG medium, as previously described (32). BMD macrophages were obtained from 6-to-8-week-old A/J mice (Jackson Laboratory) as previously described (96). BMD macrophages from C57BL/6 mice were obtained from 12-week-old mice in an identical manner. PBMCs were obtained from healthy human volunteers and cultured as previously described (97). PBMCs (10 × 10^6^) were differentiated in 10-cm-diameter dishes using 10 ml RPMI medium supplemented with 15% human serum type AB or RPMI medium supplemented with 10% FBS and 50 ng/ml recombinant human M-CSF for 3 days. After 2 to 3 days, fresh medium containing human serum or M-CSF was added and the cells were allowed to differentiate for an additional 3 to 4 days for a total of 6 days (23, 41). A total of 2.5 × 10^5^ differentiated macrophages or 5 × 10^5^ amoebae in a volume of 250 μl were seeded onto 12-mm-diameter coverslips (Electron Microscopy Sciences) in 24-well plates and allowed to adhere for 2 to 24 h. The monolayers were infected with *L. pneumophila* from 3-day-old BCYE plates at a multiplicity of infection (MOI) of 50 (for macrophages) or 5 (for amoebae) in 250 µl of media without FBS. The tissue culture plates were centrifuged at 250 × *g* for 5 min and floated on a 37°C water bath for 5 min to allow bacterial entry and were then washed three times with 500 μl media each time to remove any remaining extracellular bacteria (21). For WT 130b carrying pM*proA* and pM*chiA*, IPTG was added to the well at a final concentration of 1 mM to induce protein expression. Infection was allowed to proceed to the indicated time points in a humidified incubator at 37°C with 5% CO_2_ (for macrophages) or at 35°C (for amoebae).

### Immunofluorescence assays and microscopy.

Uninfected and infected cells were processed for immunofluorescence analysis (98). The cells were fixed in 4% (vol/vol) paraformaldehyde (Electron Microscopy Sciences) for 20 min at room temperature followed by permeabilization with 0.5% (vol/vol) TX-100 for 10 min, with ice-cold MeOH for 30 s, or with 55 μg/ml digitonin for 5 min at 4°C (99, 100). The cells were blocked for 1 h at 37°C in 5% (vol/vol) bovine serum albumin (BSA)–PBS. The T2S substrate rabbit polyclonal antisera were diluted in 250 µl 1% BSA–PBS to their working concentrations as follows: for rabbit anti-ProA, 1:400; for rabbit anti-CelA, 1:100; and for rabbit anti-ChiA, 1:200. They were then incubated in two successive wells of fixed and permeabilized uninfected macrophages or amoebae at 37°C for 1 h each time to preadsorb the antisera and reduce background. Per the recommendations of the manufacturers, rabbit anti-galectin 3 from Santa Cruz Biotechnology was used at a concentration of 1:50, rabbit anti-galectin-3 antibody from Abcam, Inc., was used at 1:250, and goat anti-galectin 8 from R&D Systems was used at 15 µg/ml. Mouse anti-LPS (MAb 3/1) antiserum was added to the 250 μl of preadsorbed antisera or to the galectin dilution at a final concentration of 1:1,500. The cells were then incubated in primary antisera for 2 h at 37°C followed by three washes with 500 μl of PBS. Secondary antibodies Alexa Fluor Oregon green-conjugated goat anti-mouse IgG antibody (Invitrogen), Alexa Fluor 488-conjugated rabbit anti-mouse IgG antibody (Invitrogen), Alexa Fluor 594-conjugated goat anti-rabbit IgG antibody (Invitrogen), and Alexa Fluor 594-conjugated rabbit anti-goat IgG antibody (Invitrogen) were each added to the cells at a concentration of 1:500 in 250 μl of 1% BSA–PBS for 1 h at 37°C. The coverslips were washed three times with 500 μl of PBS prior to mounting on slides with ProLong Gold Antifade with 4′,6-diamidino-2-phenylindole (DAPI) (Molecular Probes). Images were obtained using a Nikon C2+ or Nikon A1R laser scanning confocal microscope. To quantify localization to the macrophage cytosol and LCVM, 100 cells from each of 3 replicate experiments were analyzed on an EVOS XL cell imaging system (Thermo Fisher Scientific).

### Analysis of free LCVs.

Differentiated U937 cells (3 × 10^6^) were seeded onto four wells of a 6-well plate and allowed to adhere. The cells were infected as described above. After 16 h, the wells were washed in 2 ml of PBS and gently scraped with a cell scraper (Falcon) to remove cells. Free LCVs were prepared as previously described (36, 69). The cells were pelleted at 233 × *g* for 5 min at 4°C and resuspended in 1 ml of ice-cold homogenization hypo-osmotic buffer (20 mM HEPES–KOH, pH 7.2, 250 mM sucrose, 5 mM EGTA) with cOmplete protease inhibitor cocktail. The cells were then added to a type B Dounce homogenizer (Kimble Chase) and subjected to Dounce homogenization 5 times. Lysis of >90% of the cells was verified by trypan blue exclusion assay (101). The LCVs were separated from intact host cells and nuclei by centrifugation at 524 × *g* for 3 min at 4°C. The supernatant (500 μl) was centrifuged at 1,455 × *g* for 5 min onto poly-l-lysine-coated coverslips in a 24-well plate. The plate was incubated for 15 min at 37°C in a buffered, humidified chamber to help facilitate adhesion. The free LCVs were then fixed in 4% paraformaldehyde and analyzed by indirect immunofluorescence assay and confocal microscopy as detailed above.

## References

[1] van Heijnsbergen E, Schalk JA, Euser SM, Brandsema PS, den Boer JW, de Roda Husman AM 2015 Confirmed and potential sources of *Legionella* reviewed. Environ Sci Technol 49:4797–4815. doi:10.1021/acs.est.5b00142. 25774976

[2] Newton HJ, Ang DK, van Driel IR, Hartland EL 2010 Molecular pathogenesis of infections caused by *Legionella pneumophila*. Clin Microbiol Rev 23:274–298. doi:10.1128/CMR.00052-09. 20375353 PMC2863363

[3] Fields BS, Benson RF, Besser RE 2002 *Legionella* and Legionnaires’ disease: 25 years of investigation. Clin Microbiol Rev 15:506–526. doi:10.1128/CMR.15.3.506-526.2002. 12097254 PMC118082

[4] Edelstein PH, Cianciotto NP 2010 Legionella, p 2969–2984. *In* Mandell GL, Bennett JE, Dolin R (ed), Principles and practice of infectious diseases, 7th ed, vol. 2 Elsevier/Churchill Livingstone, Philadelphia, PA.

[5] Centers for Disease Control and Prevention (CDC) 2011 Legionellosis—United States, 2000–2009. MMWR Morb Mortal Wkly Rep 60:1083–1086. 21849965

[6] Parr A, Whitney EA, Berkelman RL 2015 Legionellosis on the rise: a review of guidelines for prevention in the United States. J Public Health Manag Pract 21:E17–E26. doi:10.1097/PHH.0000000000000123. 25203696 PMC4519350

[7] Swanson MS, Isberg RR 1995 Association of *Legionella pneumophila* with the macrophage endoplasmic reticulum. Infect Immun 63:3609–3620. 7642298 10.1128/iai.63.9.3609-3620.1995 PMC173501

[8] Roy CR, Tilney LG 2002 The road less traveled: transport of *Legionella* to the endoplasmic reticulum. J Cell Biol 158:415–419. doi:10.1083/jcb.200205011. 12147677 PMC2173838

[9] Machner MP, Isberg RR 2006 Targeting of host Rab GTPase function by the intravacuolar pathogen *Legionella pneumophila*. Dev Cell 11:47–56. doi:10.1016/j.devcel.2006.05.013. 16824952

[10] Murata T, Delprato A, Ingmundson A, Toomre DK, Lambright DG, Roy CR 2006 The *Legionella pneumophila* effector protein DrrA is a Rab1 guanine nucleotide-exchange factor. Nat Cell Biol 8:971–977. doi:10.1038/ncb1463. 16906144

[11] Swanson MS, Bachman MA 2002 The *Legionella pneumophila* life cycle: connections between growth phase, virulence expression, and replication vacuole biogenesis, p 74–81. *In* Marre R, Kwaik YA, Bartlett C, Cianciotto NP, Fields BS, Frosch M, Hacker J, Lück PC (ed), Legionella. ASM Press, Washington, DC.

[12] Isberg RR, O’Connor TJ, Heidtman M 2009 The *Legionella pneumophila* replication vacuole: making a cosy niche inside host cells. Nat Rev Microbiol 7:13–24. doi:10.1038/nrmicro1967. 19011659 PMC2631402

[13] Hubber A, Roy CR 2010 Modulation of host cell function by *Legionella pneumophila* type IV effectors. Annu Rev Cell Dev Biol 26:261–283. doi:10.1146/annurev-cellbio-100109-104034. 20929312

[14] de Felipe KS, Glover RT, Charpentier X, Anderson OR, Reyes M, Pericone CD, Shuman HA 2008 *Legionella* eukaryotic-like type IV substrates interfere with organelle trafficking. PLoS Pathog 4:e1000117. doi:10.1371/journal.ppat.1000117. 18670632 PMC2475511

[15] Urbanus ML, Quaile AT, Stogios PJ, Morar M, Rao C, Di Leo R, Evdokimova E, Lam M, Oatway C, Cuff ME, Osipiuk J, Michalska K, Nocek BP, Taipale M, Savchenko A, Ensminger AW 2016 Diverse mechanisms of metaeffector activity in an intracellular bacterial pathogen, *Legionella pneumophila*. Mol Syst Biol 12:893. doi:10.15252/msb.20167381. 27986836 PMC5199130

[16] Cianciotto NP 2005 Type II secretion: a protein secretion system for all seasons. Trends Microbiol 13:581–588. doi:10.1016/j.tim.2005.09.005. 16216510

[17] Hales LM, Shuman HA 1999 *Legionella pneumophila* contains a type II general secretion pathway required for growth in amoebae as well as for secretion of the Msp protease. Infect Immun 67:3662–3666. 10377156 10.1128/iai.67.7.3662-3666.1999 PMC116561

[18] Cianciotto NP, White RC 2017 Expanding role of type II secretion in bacterial pathogenesis and beyond. Infect Immun 85:e00014-17. doi:10.1128/IAI.00014-17. 28264910 PMC5400843

[19] Söderberg MA, Dao J, Starkenburg SR, Cianciotto NP 2008 Importance of type II secretion for survival of *Legionella pneumophila* in tap water and in amoebae at low temperatures. Appl Environ Microbiol 74:5583–5588. doi:10.1128/AEM.00067-08. 18621869 PMC2546640

[20] Rossier O, Starkenburg SR, Cianciotto NP 2004 *Legionella pneumophila* type II protein secretion promotes virulence in the A/J mouse model of Legionnaires’ disease pneumonia. Infect Immun 72:310–321. doi:10.1128/IAI.72.1.310-321.2004. 14688110 PMC344012

[21] White RC, Cianciotto NP 2016 Type II secretion is necessary for the optimal association of the *Legionella*-containing vacuole with macrophage Rab1B but mainly enhances intracellular replication by Rab1B-independent mechanisms. Infect Immun 84:3313–3327. doi:10.1128/IAI.00750-16. 27600508 PMC5116710

[22] Roy CR 2012 Vacuolar pathogens value membrane integrity. Proc Natl Acad Sci U S A 109:3197–3198. doi:10.1073/pnas.1200326109. 22343287 PMC3295304

[23] Mallama CA, McCoy-Simandle K, Cianciotto NP 2017 The type II secretion system of *Legionella pneumophila* dampens the MyD88 and Toll-like receptor 2 signaling pathway in infected human macrophages. Infect Immun 85:e00897-16. doi:10.1128/IAI.00897-16. 28138020 PMC5364298

[24] McCoy-Simandle K, Stewart CR, Dao J, DebRoy S, Rossier O, Bryce PJ, Cianciotto NP 2011 *Legionella pneumophila* type II secretion dampens the cytokine response of infected macrophages and epithelia. Infect Immun 79:1984–1997. doi:10.1128/IAI.01077-10. 21383054 PMC3088156

[25] Conlan JW, Williams A, Ashworth LA 1988 In vivo production of a tissue-destructive protease by *Legionella pneumophila* in the lungs of experimentally infected guinea-pigs. J Gen Microbiol 134:143–149. doi:10.1099/00221287-134-1-143. 3053969

[26] Rechnitzer C, Williams A, Wright JB, Dowsett AB, Milman N, Fitzgeorge RB 1992 Demonstration of the intracellular production of tissue-destructive protease by *Legionella pneumophila* multiplying within guinea-pig and human alveolar macrophages. J Gen Microbiol 138:1671–1677. doi:10.1099/00221287-138-8-1671. 1527507

[27] Moffat JF, Edelstein PH, Regula DP Jr, Cirillo JD, Tompkins LS 1994 Effects of an isogenic Zn-metalloprotease-deficient mutant of *Legionella pneumophila* in a guinea-pig pneumonia model. Mol Microbiol 12:693–705. doi:10.1111/j.1365-2958.1994.tb01057.x. 8052122

[28] Quinn FD, Tompkins LS 1989 Analysis of a cloned sequence of *Legionella pneumophila* encoding a 38 kD metalloprotease possessing haemolytic and cytotoxic activities. Mol Microbiol 3:797–805. doi:10.1111/j.1365-2958.1989.tb00228.x. 2546010

[29] Keen MG, Hoffman PS 1989 Characterization of a *Legionella pneumophila* extracellular protease exhibiting hemolytic and cytotoxic activities. Infect Immun 57:732–738. 2917785 10.1128/iai.57.3.732-738.1989 PMC313170

[30] Szeto L, Shuman HA 1990 The *Legionella pneumophila* major secretory protein, a protease, is not required for intracellular growth or cell killing. Infect Immun 58:2585–2592. 2164510 10.1128/iai.58.8.2585-2592.1990 PMC258859

[31] Rossier O, Dao J, Cianciotto NP 2008 The type II secretion system of *Legionella pneumophila* elaborates two aminopeptidases, as well as a metalloprotease that contributes to differential infection among protozoan hosts. Appl Environ Microbiol 74:753–761. doi:10.1128/AEM.01944-07. 18083880 PMC2227731

[32] Tyson JY, Pearce MM, Vargas P, Bagchi S, Mulhern BJ, Cianciotto NP 2013 Multiple *Legionella pneumophila* type II secretion substrates, including a novel protein, contribute to differential infection of the amoebae *Acanthamoeba castellanii*, *Hartmannella vermiformis*, and *Naegleria lovaniensis*. Infect Immun 81:1399–1410. doi:10.1128/IAI.00045-13. 23429532 PMC3648003

[33] Reichardt K, Jacobs E, Röske I, Helbig JH 2010 *Legionella pneumophila* carrying the virulence-associated lipopolysaccharide epitope possesses two functionally different LPS components. Microbiology 156:2953–2961. doi:10.1099/mic.0.039933-0. 20656784

[34] Jamur MC, Oliver C 2010 Permeabilization of cell membranes. Methods Mol Biol 588:63–66. doi:10.1007/978-1-59745-324-0_9. 20012820

[35] Liu Q, Pante N, Misteli T, Elsagga M, Crisp M, Hodzic D, Burke B, Roux KJ 2007 Functional association of Sun1 with nuclear pore complexes. J Cell Biol 178:785–798. doi:10.1083/jcb.200704108. 17724119 PMC2064544

[36] Price CT, Al-Khodor S, Al-Quadan T, Santic M, Habyarimana F, Kalia A, Kwaik YA 2009 Molecular mimicry by an F-box effector of *Legionella pneumophila* hijacks a conserved polyubiquitination machinery within macrophages and protozoa. PLoS Pathog 5:e1000704. doi:10.1371/journal.ppat.1000704. 20041211 PMC2790608

[37] Mia S, Warnecke A, Zhang XM, Malmström V, Harris RA 2014 An optimized protocol for human M2 macrophages using M-CSF and IL-4/IL-10/TGF-beta yields a dominant immunosuppressive phenotype. Scand J Immunol 79:305–314. doi:10.1111/sji.12162. 24521472 PMC4282403

[38] Saghaeian-Jazi M, Mohammadi S, Sedighi S 2016 Culture and differentiation of monocyte derived macrophages using human serum: an optimized method. Zahedan J Res Med Sci doi:10.17795/zjrms-7362.

[39] Jung AL, Stoiber C, Herkt CE, Schulz C, Bertrams W, Schmeck B 2016 *Legionella pneumophila*-derived outer membrane vesicles promote bacterial replication in macrophages. PLoS Pathog 12:e1005592. doi:10.1371/journal.ppat.1005592. 27105429 PMC4841580

[40] Faucher SP, Mueller CA, Shuman HA 2011 *Legionella pneumophila* transcriptome during intracellular multiplication in human macrophages. Front Microbiol 2:60. doi:10.3389/fmicb.2011.00060. 21747786 PMC3128937

[41] Casson CN, Yu J, Reyes VM, Taschuk FO, Yadav A, Copenhaver AM, Nguyen HT, Collman RG, Shin S 2015 Human caspase-4 mediates noncanonical inflammasome activation against gram-negative bacterial pathogens. Proc Natl Acad Sci U S A 112:6688–6693. doi:10.1073/pnas.1421699112. 25964352 PMC4450384

[42] Cianciotto NP 2013 Type II secretion and *Legionella* virulence. Curr Top Microbiol Immunol 376:81–102. doi:10.1007/82_2013_339. 23900831

[43] Fortier A, Faucher SP, Diallo K, Gros P 2011 Global cellular changes induced by *Legionella pneumophila* infection of bone marrow-derived macrophages. Immunobiology 216:1274–1285. doi:10.1016/j.imbio.2011.06.008. 21794945

[44] Jayakumar D, Early JV, Steinman HM 2012 Virulence phenotypes of *Legionella pneumophila* associated with noncoding RNA lpr0035. Infect Immun 80:4143–4153. doi:10.1128/IAI.00598-12. 22966048 PMC3497406

[45] Isaac DT, Laguna RK, Valtz N, Isberg RR 2015 MavN is a *Legionella pneumophila* vacuole-associated protein required for efficient iron acquisition during intracellular growth. Proc Natl Acad Sci U S A 112:E5208–E5217. doi:10.1073/pnas.1511389112. PMC4577140 26330609

[46] Molofsky AB, Byrne BG, Whitfield NN, Madigan CA, Fuse ET, Tateda K, Swanson MS 2006 Cytosolic recognition of flagellin by mouse macrophages restricts *Legionella pneumophila* infection. J Exp Med 203:1093–1104. doi:10.1084/jem.20051659. 16606669 PMC1584282

[47] Lightfield KL, Persson J, Brubaker SW, Witte CE, von Moltke J, Dunipace EA, Henry T, Sun YH, Cado D, Dietrich WF, Monack DM, Tsolis RM, Vance RE 2008 Critical function for Naip5 in inflammasome activation by a conserved carboxy-terminal domain of flagellin. Nat Immunol 9:1171–1178. doi:10.1038/ni.1646. 18724372 PMC2614210

[48] Mercante JW, Morrison SS, Desai HP, Raphael BH, Winchell JM 2016 Genomic analysis reveals novel diversity among the 1976 Philadelphia Legionnaires’ disease outbreak isolates and additional ST36 strains. PLoS One 11:e0164074. doi:10.1371/journal.pone.0164074. 27684472 PMC5042515

[49] Ensminger AW 2016 *Legionella pneumophila*, armed to the hilt: justifying the largest arsenal of effectors in the bacterial world. Curr Opin Microbiol 29:74–80. doi:10.1016/j.mib.2015.11.002. 26709975

[50] Fuche F, Vianney A, Andrea C, Doublet P, Gilbert C 2015 Functional type 1 secretion system involved in *Legionella pneumophila* virulence. J Bacteriol 197:563–571. doi:10.1128/JB.02164-14. 25422301 PMC4285970

[51] Söderberg MA, Cianciotto NP 2008 A *Legionella pneumophila* peptidyl-prolyl *cis*-*trans* isomerase present in culture supernatants is necessary for optimal growth at low temperatures. Appl Environ Microbiol 74:1634–1638. doi:10.1128/AEM.02512-07. 18165359 PMC2258609

[52] Roy CR, Isberg RR 1997 Topology of *Legionella pneumophila* DotA: an inner membrane protein required for replication in macrophages. Infect Immun 65:571–578. 9009315 10.1128/iai.65.2.571-578.1997 PMC176098

[53] Berger KH, Merriam JJ, Isberg RR 1994 Altered intracellular targeting properties associated with mutations in the *Legionella pneumophila* dotA gene. Mol Microbiol 14:809–822. doi:10.1111/j.1365-2958.1994.tb01317.x. 7891566

[54] Molmeret M, Santic’ M, Asare R, Carabeo RA, Abu Kwaik Y 2007 Rapid escape of the dot/icm mutants of *Legionella pneumophila* into the cytosol of mammalian and protozoan cells. Infect Immun 75:3290–3304. doi:10.1128/IAI.00292-07. 17438033 PMC1932949

[55] Ninio S, Roy CR 2007 Effector proteins translocated by *Legionella pneumophila*: strength in numbers. Trends Microbiol 15:372–380. doi:10.1016/j.tim.2007.06.006. 17632005

[56] Ensminger AW, Isberg RR 2009 *Legionella pneumophila* Dot/Icm translocated substrates: a sum of parts. Curr Opin Microbiol 12:67–73. doi:10.1016/j.mib.2008.12.004. 19157961 PMC2741304

[57] Burstein D, Zusman T, Degtyar E, Viner R, Segal G, Pupko T 2009 Genome-scale identification of *Legionella pneumophila* effectors using a machine learning approach. PLoS Pathog 5:e1000508. doi:10.1371/journal.ppat.1000508. 19593377 PMC2701608

[58] Segal G 2013 Identification of legionella effectors using bioinformatic approaches. Methods Mol Biol 954:595–602. doi:10.1007/978-1-62703-161-5_37. 23150423

[59] DebRoy S, Dao J, Söderberg M, Rossier O, Cianciotto NP 2006 *Legionella pneumophila* type II secretome reveals unique exoproteins and a chitinase that promotes bacterial persistence in the lung. Proc Natl Acad Sci U S A 103:19146–19151. doi:10.1073/pnas.0608279103. 17148602 PMC1748190

[60] Galka F, Wai SN, Kusch H, Engelmann S, Hecker M, Schmeck B, Hippenstiel S, Uhlin BE, Steinert M 2008 Proteomic characterization of the whole secretome of *Legionella pneumophila* and functional analysis of outer membrane vesicles. Infect Immun 76:1825–1836. doi:10.1128/IAI.01396-07. 18250176 PMC2346698

[61] Kuroda T, Kubori T, Thanh Bui X, Hyakutake A, Uchida Y, Imada K, Nagai H 2015 Molecular and structural analysis of *Legionella* DotI gives insights into an inner membrane complex essential for type IV secretion. Sci Rep 5:10912. doi:10.1038/srep10912. 26039110 PMC4454188

[62] Paz I, Sachse M, Dupont N, Mounier J, Cederfur C, Enninga J, Leffler H, Poirier F, Prevost MC, Lafont F, Sansonetti P 2010 Galectin-3, a marker for vacuole lysis by invasive pathogens. Cell Microbiol 12:530–544. doi:10.1111/j.1462-5822.2009.01415.x. 19951367

[63] Machado FC, Cruz L, da Silva AA, Cruz MC, Mortara RA, Roque-Barreira MC, da Silva CV 2014 Recruitment of galectin-3 during cell invasion and intracellular trafficking of *Trypanosoma cruzi* extracellular amastigotes. Glycobiology 24:179–184. doi:10.1093/glycob/cwt097. 24225883

[64] Barboni E, Coade S, Fiori A 2005 The binding of mycolic acids to galectin-3: a novel interaction between a host soluble lectin and trafficking mycobacterial lipids? FEBS Lett 579:6749–6755. doi:10.1016/j.febslet.2005.11.005. 16310777

[65] Mey A, Leffler H, Hmama Z, Normier G, Revillard JP 1996 The animal lectin galectin-3 interacts with bacterial lipopolysaccharides via two independent sites. J Immunol 156:1572–1577. 8568262

[66] Gupta SK, Masinick S, Garrett M, Hazlett LD 1997 *Pseudomonas aeruginosa* lipopolysaccharide binds galectin-3 and other human corneal epithelial proteins. Infect Immun 65:2747–2753. 9199445 10.1128/iai.65.7.2747-2753.1997 PMC175387

[67] Thurston TL, Wandel MP, von Muhlinen N, Foeglein A, Randow F 2012 Galectin 8 targets damaged vesicles for autophagy to defend cells against bacterial invasion. Nature 482:414–418. doi:10.1038/nature10744. 22246324 PMC3343631

[68] Cianciotto NP 2009 Many substrates and functions of type II secretion: lessons learned from *Legionella pneumophila*. Future Microbiol 4:797–805. doi:10.2217/fmb.09.53. 19722835 PMC2754693

[69] Creasey EA, Isberg RR 2012 The protein SdhA maintains the integrity of the *Legionella*-containing vacuole. Proc Natl Acad Sci U S A 109:3481–3486. doi:10.1073/pnas.1121286109. 22308473 PMC3295292

[70] Feeley EM, Pilla-Moffett DM, Zwack EE, Piro AS, Finethy R, Kolb JP, Martinez J, Brodsky IE, Coers J 2017 Galectin-3 directs antimicrobial guanylate binding proteins to vacuoles furnished with bacterial secretion systems. Proc Natl Acad Sci U S A 114:E1698–E1706. doi:10.1073/pnas.1615771114. PMC5338555 28193861

[71] Robinson CG, Roy CR 2006 Attachment and fusion of endoplasmic reticulum with vacuoles containing *Legionella pneumophila*. Cell Microbiol 8:793–805. doi:10.1111/j.1462-5822.2005.00666.x. 16611228

[72] Wieland H, Ullrich S, Lang F, Neumeister B 2005 Intracellular multiplication of *Legionella pneumophila* depends on host cell amino acid transporter SLC1A5. Mol Microbiol 55:1528–1537. doi:10.1111/j.1365-2958.2005.04490.x. 15720558

[73] Bruckert WM, Abu Kwaik Y 2015 Complete and ubiquitinated proteome of the *Legionella*-containing vacuole within human macrophages. J Proteome Res 14:236–248. doi:10.1021/pr500765x. 25369898 PMC4286187

[74] Teitelbaum R, Cammer M, Maitland ML, Freitag NE, Condeelis J, Bloom BR 1999 Mycobacterial infection of macrophages results in membrane-permeable phagosomes. Proc Natl Acad Sci U S A 96:15190–15195. doi:10.1073/pnas.96.26.15190. 10611360 PMC24795

[75] Grotzke JE, Harriff MJ, Siler AC, Nolt D, Delepine J, Lewinsohn DA, Lewinsohn DM 2009 The *Mycobacterium tuberculosis* phagosome is a HLA-I processing competent organelle. PLoS Pathog 5:e1000374. doi:10.1371/journal.ppat.1000374. 19360129 PMC2661020

[76] Harriff MJ, Burgdorf S, Kurts C, Wiertz EJ, Lewinsohn DA, Lewinsohn DM 2013 TAP mediates import of *Mycobacterium tuberculosis*-derived peptides into phagosomes and facilitates loading onto HLA-I. PLoS One 8:e79571. doi:10.1371/journal.pone.0079571. 24244525 PMC3823705

[77] Rosales-Reyes R, Aubert DF, Tolman JS, Amer AO, Valvano MA 2012 *Burkholderia cenocepacia* type VI secretion system mediates escape of type II secreted proteins into the cytoplasm of infected macrophages. PLoS One 7:e41726. doi:10.1371/journal.pone.0041726. 22848580 PMC3405007

[78] London N, Lamphear CL, Hougland JL, Fierke CA, Schueler-Furman O 2011 Identification of a novel class of farnesylation targets by structure-based modeling of binding specificity. PLoS Comput Biol 7:e1002170. doi:10.1371/journal.pcbi.1002170. 21998565 PMC3188499

[79] Black WJ, Quinn FD, Tompkins LS 1990 *Legionella pneumophila* zinc metalloprotease is structurally and functionally homologous to *Pseudomonas aeruginosa* elastase. J Bacteriol 172:2608–2613. doi:10.1128/jb.172.5.2608-2613.1990. 2110146 PMC208904

[80] Ivanov SS, Roy C 2013 Host lipidation: a mechanism for spatial regulation of *Legionella* effectors. Curr Top Microbiol Immunol 376:135–154. doi:10.1007/82_2013_344. 23918175

[81] Price CT, Al-Quadan T, Santic M, Jones SC, Abu Kwaik Y 2010 Exploitation of conserved eukaryotic host cell farnesylation machinery by an F-box effector of *Legionella pneumophila*. J Exp Med 207:1713–1726. doi:10.1084/jem.20100771. 20660614 PMC2916131

[82] Schnell U, Dijk F, Sjollema KA, Giepmans BN 2012 Immunolabeling artifacts and the need for live-cell imaging. Nat Methods 9:152–158. doi:10.1038/nmeth.1855. 22290187

[83] Ninio S, Celli J, Roy CR 2009 A *Legionella pneumophila* effector protein encoded in a region of genomic plasticity binds to Dot/Icm-modified vacuoles. PLoS Pathog 5:e1000278. doi:10.1371/journal.ppat.1000278. 19165328 PMC2621349

[84] Campodonico EM, Chesnel L, Roy CR 2005 A yeast genetic system for the identification and characterization of substrate proteins transferred into host cells by the *Legionella pneumophila* Dot/Icm system. Mol Microbiol 56:918–933. doi:10.1111/j.1365-2958.2005.04595.x. 15853880

[85] Brombacher E, Urwyler S, Ragaz C, Weber SS, Kami K, Overduin M, Hilbi H 2009 Rab1 guanine nucleotide exchange factor SidM is a major phosphatidylinositol 4-phosphate-binding effector protein of *Legionella pneumophila*. J Biol Chem 284:4846–4856. doi:10.1074/jbc.M807505200. 19095644 PMC2643517

[86] Haneburger I, Hilbi H 2013 Phosphoinositide lipids and the *Legionella* pathogen vacuole. Curr Top Microbiol Immunol 376:155–173. doi:10.1007/82_2013_341. 23918172

[87] Conlan JW, Baskerville A, Ashworth LA 1986 Separation of *Legionella pneumophila* proteases and purification of a protease which produces lesions like those of Legionnaires’ disease in guinea pig lung. J Gen Microbiol 132:1565–1574. doi:10.1099/00221287-132-6-1565. 3543209

[88] Müller MP, Shkumatov AV, Oesterlin LK, Schoebel S, Goody PR, Goody RS, Itzen A 2012 Characterization of enzymes from *Legionella pneumophila* involved in reversible adenylylation of Rab1 protein. J Biol Chem 287:35036–35046. doi:10.1074/jbc.M112.396861. 22872634 PMC3471704

[89] Ingmundson A, Delprato A, Lambright DG, Roy CR 2007 *Legionella pneumophila* proteins that regulate Rab1 membrane cycling. Nature 450:365–369. doi:10.1038/nature06336. 17952054

[90] Rao FV, Dorfmueller HC, Villa F, Allwood M, Eggleston IM, van Aalten DMF 2006 Structural insights into the mechanism and inhibition of eukaryotic O-GlcNAc hydrolysis. EMBO J 25:1569–1578. doi:10.1038/sj.emboj.7601026. 16541109 PMC1440316

[91] O’Connor TJ, Adepoju Y, Boyd D, Isberg RR 2011 Minimization of the *Legionella pneumophila* genome reveals chromosomal regions involved in host range expansion. Proc Natl Acad Sci U S A 108:14733–14740. doi:10.1073/pnas.1111678108. 21873199 PMC3169125

[92] Cianciotto NP, Eisenstein BI, Mody CH, Toews GB, Engleberg NC 1989 A *Legionella pneumophila* gene encoding a species-specific surface protein potentiates initiation of intracellular infection. Infect Immun 57:1255–1262. 2925251 10.1128/iai.57.4.1255-1262.1989 PMC313258

[93] Pearce MM, Cianciotto NP 2009 *Legionella pneumophila* secretes an endoglucanase that belongs to the family-5 of glycosyl hydrolases and is dependent upon type II secretion. FEMS Microbiol Lett 300:256–264. doi:10.1111/j.1574-6968.2009.01801.x. 19817866 PMC2766432

[94] Stewart CR, Burnside DM, Cianciotto NP 2011 The surfactant of *Legionella pneumophila* is secreted in a TolC-dependent manner and is antagonistic toward other *Legionella* species. J Bacteriol 193:5971–5984. doi:10.1128/JB.05405-11. 21890700 PMC3194911

[95] Stewart CR, Rossier O, Cianciotto NP 2009 Surface translocation by *Legionella pneumophila*: a form of sliding motility that is dependent upon type II protein secretion. J Bacteriol 191:1537–1546. doi:10.1128/JB.01531-08. 19114479 PMC2648193

[96] Gunderson FF, Cianciotto NP 2013 The CRISPR-associated gene cas2 of *Legionella pneumophila* is required for intracellular infection of amoebae. mBio 4:e00074-13. doi:10.1128/mBio.00074-13. 23481601 PMC3604779

[97] Muller WA, Luscinskas FW 2008 Assays of transendothelial migration in vitro. Methods Enzymol 443:155–176. doi:10.1016/S0076-6879(08)02009-0. 18772016 PMC2759874

[98] Beyer AR, Truchan HK, May LJ, Walker NJ, Borjesson DL, Carlyon JA 2015 The *Anaplasma phagocytophilum* effector AmpA hijacks host cell SUMOylation. Cell Microbiol 17:504–519. doi:10.1111/cmi.12380. 25308709 PMC4664186

[99] Derré I, Isberg RR 2005 LidA, a translocated substrate of the *Legionella pneumophila* type IV secretion system, interferes with the early secretory pathway. Infect Immun 73:4370–4380. doi:10.1128/IAI.73.7.4370-4380.2005. 15972532 PMC1168608

[100] Lee VT, Anderson DM, Schneewind O 1998 Targeting of *Yersinia* Yop proteins into the cytosol of HeLa cells: one-step translocation of YopE across bacterial and eukaryotic membranes is dependent on SycE chaperone. Mol Microbiol 28:593–601. doi:10.1046/j.1365-2958.1998.00822.x. 9632261

[101] Strober W 2001 Trypan blue exclusion test of cell viability. Curr Protoc Immunol 21:3B:A.3B.1–A.3B.2. doi:10.1002/0471142735.ima03bs21. 18432654

