# Supplementary figures and images for: Type II Secretion Substrates of Legionella pneumophila Translocate Out of the Pathogen-Occupied Vacuole via a Semipermeable Membrane
Source: mBio. 2017 Jun 20;8(3):e00870-17. doi: 10.1128/mBio.00870-17 (PMC5478897; doi:10.1128/mBio.00870-17)

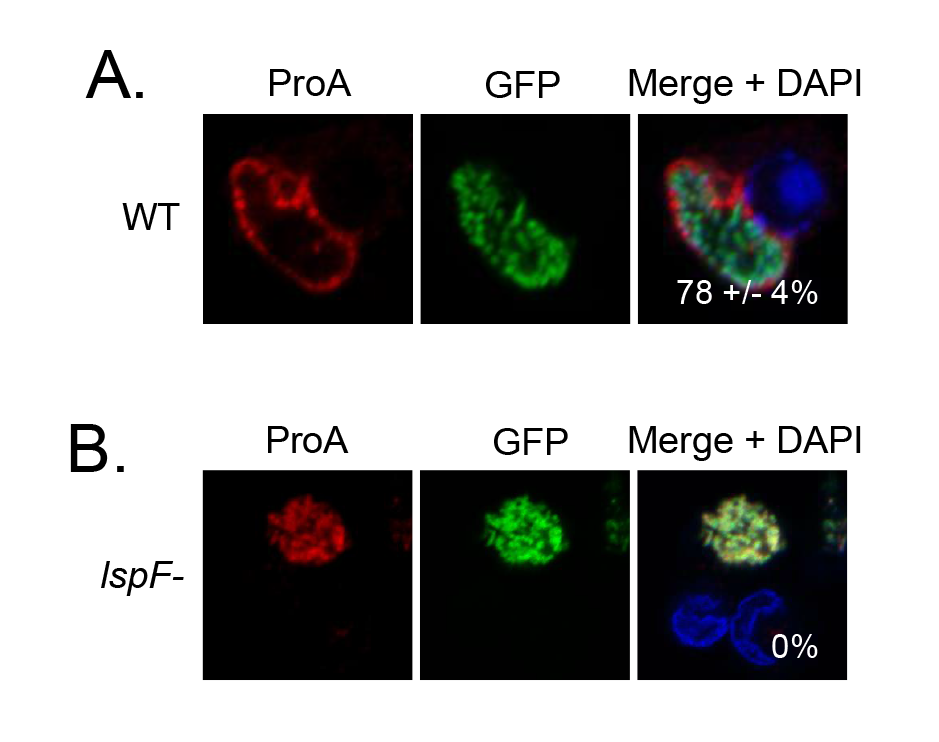

Supplement: FIG S1 [file mbo003173358sf1.tif]

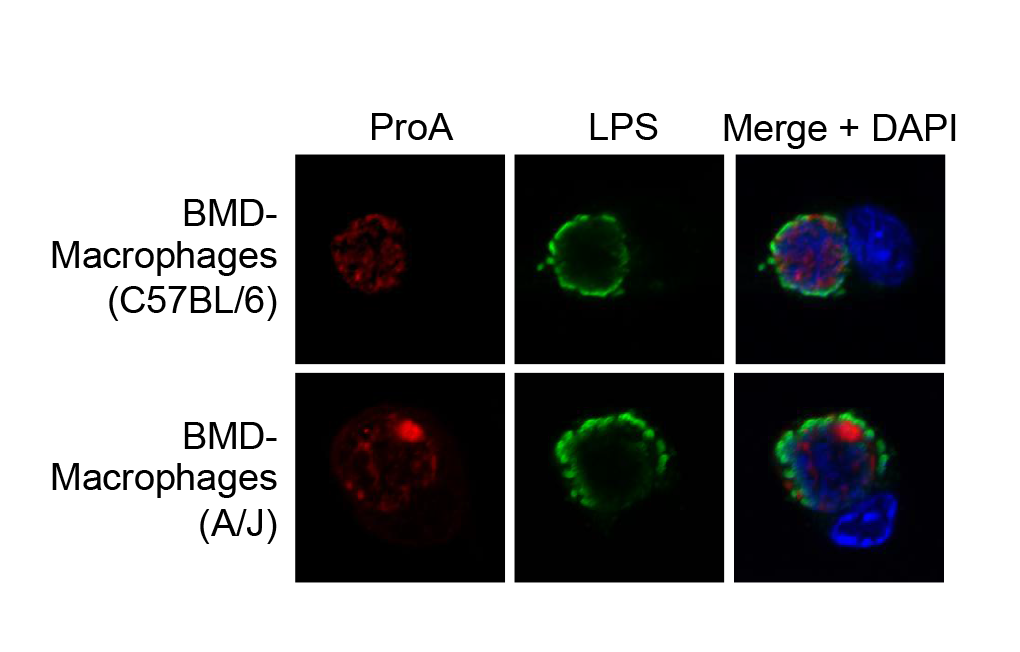

Supplement: FIG S2 [file mbo003173358sf2.tif]

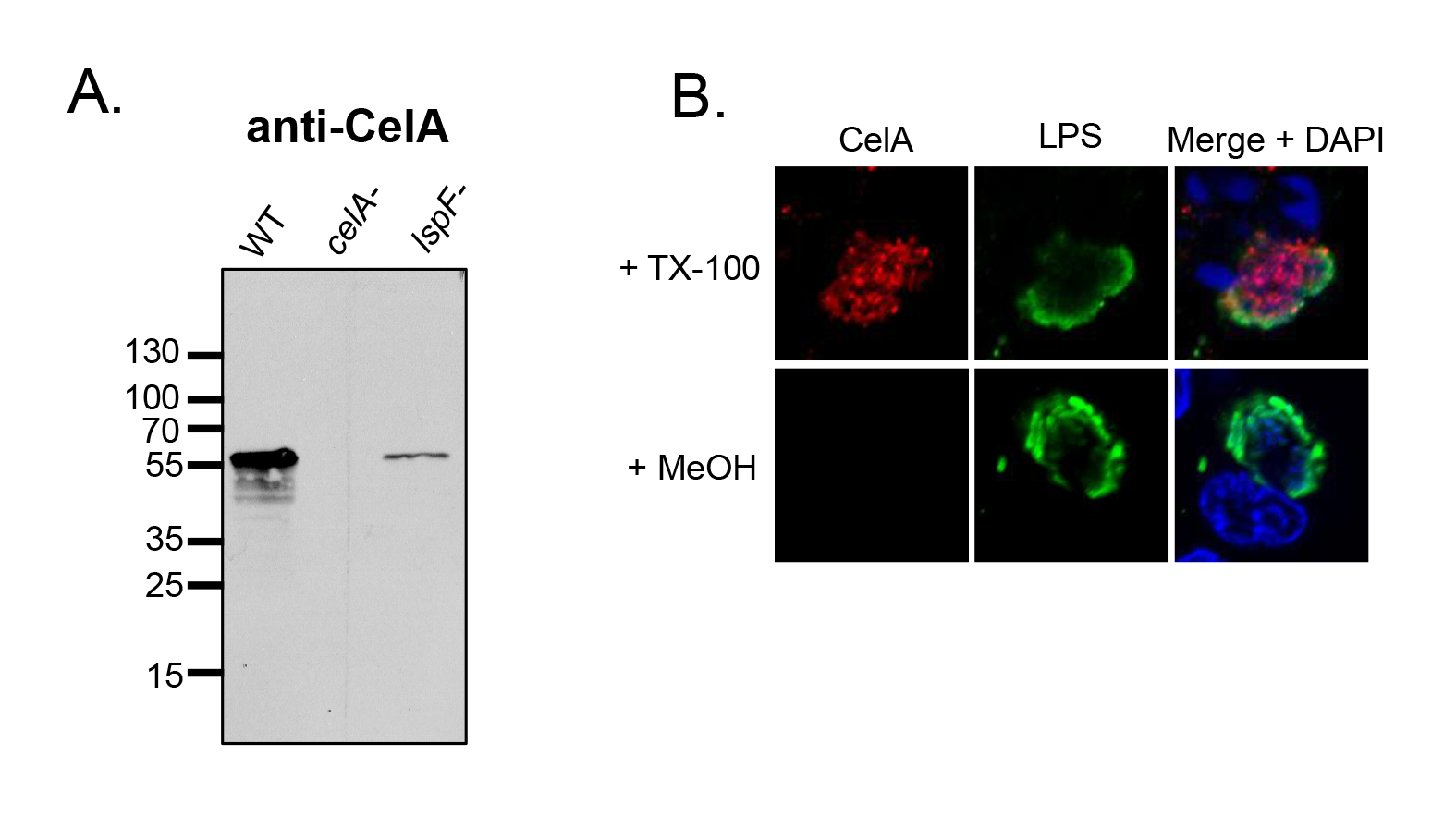

Supplement: FIG S3 [file mbo003173358sf3.tif]

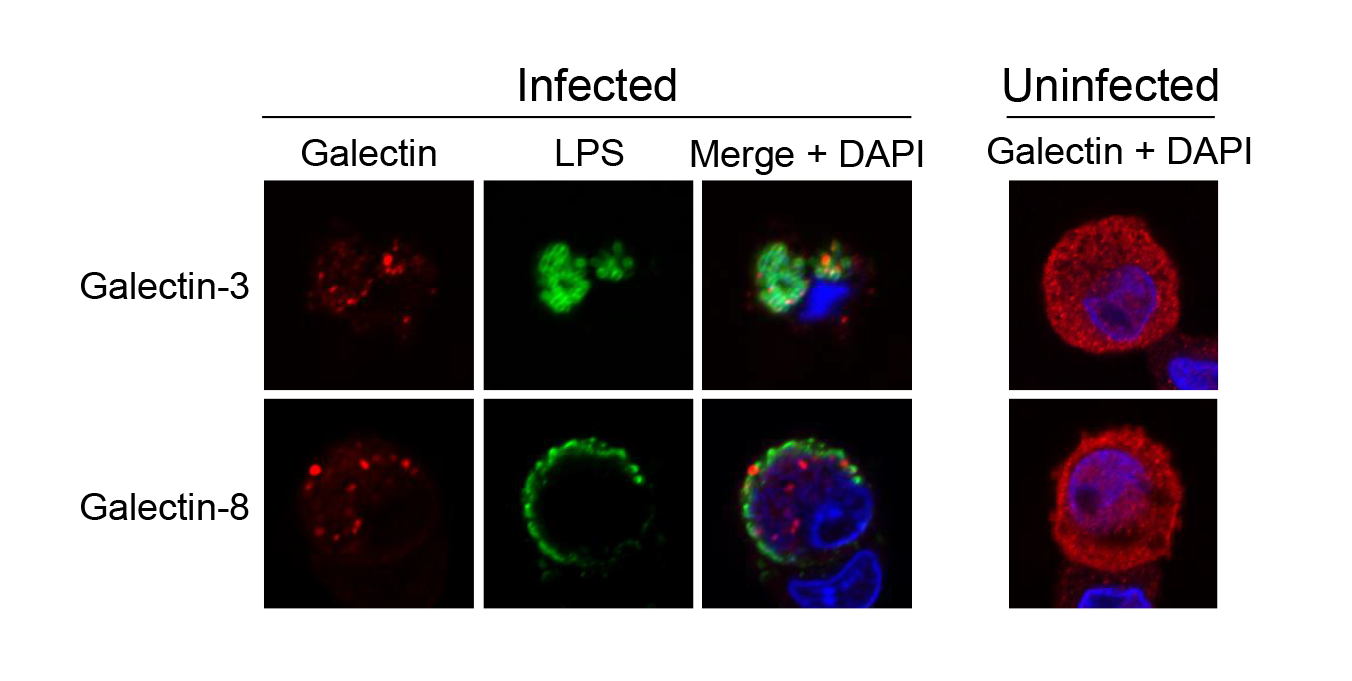

Supplement: FIG S4 [file mbo003173358sf4.tif]
